# Controlling the length of porphyrin supramolecular polymers via coupled equilibria and dilution-induced supramolecular polymerization

**DOI:** 10.1038/s41467-021-27831-2

**Published:** 2022-01-11

**Authors:** Elisabeth Weyandt, Luigi Leanza, Riccardo Capelli, Giovanni M. Pavan, Ghislaine Vantomme, E. W. Meijer

**Affiliations:** 1 Laboratory of Macromolecular and Organic Chemistry, Eindhoven University of Technology, P.O. Box 513, 5600 Eindhoven, MB The Netherlands; 2 Institute for Complex Molecular Systems (ICMS), Eindhoven University of Technology, P.O. Box 513, 5600 Eindhoven, MB The Netherlands; 3 Department of Applied Science and Technology, Politecnico di Torino, 10129 Torino, Italy; 4 Department of Innovative Technologies, University of Applied Sciences and Arts of Southern Switzerland, 6962 Lugano-Viganello, Switzerland

**Keywords:** Supramolecular polymers, Self-assembly

## Abstract

Multi-component systems often display convoluted behavior, pathway complexity and coupled equilibria. In recent years, several ways to control complex systems by manipulating the subtle balances of interaction energies between the individual components have been explored and thereby shifting the equilibrium between different aggregate states. Here we show the enantioselective chain-capping and dilution-induced supramolecular polymerization with a Zn^2+^-porphyrin-based supramolecular system when going from long, highly cooperative supramolecular polymers to short, disordered aggregates by adding a monotopic Mn^3+^-porphyrin monomer. When mixing the zinc and manganese centered monomers, the Mn^3+^-porphyrins act as chain-cappers for Zn^2+^-porphyrin supramolecular polymers, effectively hindering growth of the copolymer and reducing the length. Upon dilution, the interaction between chain-capper and monomers weakens as the equilibria shift and long supramolecular polymers form again. This dynamic modulation of aggregate morphology and length is achieved through enantioselectivity in the aggregation pathways and concentration-sensitive equilibria. All-atom and coarse-grained molecular simulations provide further insights into the mixing of the species and their exchange dynamics. Our combined experimental and theoretical approach allows for precise control of molecular self-assembly and chiral discrimination in complex systems.

## Abstract (web summary)

Controlling multicomponent systems is difficult due to convoluted behavior, pathway complexity, and coupled equilibria. Here the authors showed modulation of aggregate morphology in a zinc porphyrin-based supramolecular system via judicious capping with a manganese porphyrin monomer, in which the monomer’s chirality can influence the supramolecular behavior.

## Introduction

Porphyrin-based supramolecular systems have received great attention in recent years, due to their intriguing and often complex stereoselective assembly behavior^1^. The extended π-system surrounding the core gives rise to high levels of absorptions in the ultra-violet and visible region, but it also causes a strong drive to aggregate through π-stacking and solvophobic interactions^2^. Small changes to the structure have significant influence on the assembly behavior, such as the amide connectivity of porphyrin supramolecular monomers^3^. While *C*-centered monomers assemble into highly cooperative supramolecular polymers, their *N*-centered counterparts only form short, non-helical J-aggregates due to a high rotation barrier around the amide groups preventing the formation of hydrogen bonds. In addition, porphyrins often exhibit pathway complexity, meaning that the monomers assemble into several types of aggregates via competing pathways and mechanisms^4,5^. The manipulation of the assembly equilibria under either kinetic or thermodynamic control has resulted in the discovery of exciting phenomena. Sugiyasu and others exploited the competition between different aggregate states in a living supramolecular polymerization to obtain fibers with controlled length and narrow dispersities^6–11^. Similar approaches have been employed for seeded growth of supramolecular polymers in one or two dimensions and the preparation of supramolecular block copolymers and polymorphs under kinetic control^12–15^.

The assembly behavior of porphyrins becomes even more challenging in multi-component systems. Here not only the homo-interactions between monomers must be considered, but also the hetero-interactions between all components over a wide range of conditions such as temperature, composition, solvent, and concentration. A number of elegant examples demonstrate selectivity of one pathway over the other by small changes in the solvent composition^16–19^. The group of Aida reported on thermally bisignate polymerization of porphyrin monomers by tuning the interactions between an alcohol associating with the monomers over a wide range of temperatures^20,21^. Our own group reported dilution-induced self-assembly of a monomer in the presence of pyridine by manipulating the coupled equilibria of complexed monomer versus polymer formation over a range of concentrations^22^. For all those reasons, one-dimensional porphyrin polymers are not only of interest for organic and polymer chemists, but also to close the gap to analogous complex aggregation phenomena in natural molecular systems.

Enantioselective interactions can reduce the level of complexity in multi-component systems by introducing specificity in the interactions of the components, which can be helpful to isolate effects in the aggregation pathways. Chiral recognition and exchange of chiral information are crucial in biological systems, but also in chemical catalysis^23–26^, host-guest complexes^27–29^ and supramolecular systems^30–35^. In the supramolecular polymerizations of homochiral monomers, *M-* or *P-*helical fibers are formed. For heterochiral monomer mixtures, the monomers either follow the majority rules and intercalate into a stack of majority-preferred helicity^36,37^, form alternating heterochiral polymers^38^, or the monomers narcissistically self-sort if the mismatch penalty for co-aggregate formation is too high. Nakashima et al. reported a method to tune aggregate length and morphology by balancing the enantiomeric excess (ee) of the components, leading to heterochiral dimers in racemic mixtures or homochiral polymers at high ee’s^39^. Another remarkable example reported the use of topological chirality to achieve narcissistic self-sorting into homochiral dimers, but social self-sorting into a heterochiral polymer with alternating microstructure^40^. Seminal work by George et al. demonstrated chirality driven self-sorting and stereoselective seeded supramolecular polymerizations with a series of naphthalene diimide (NDI) functionalized monomers^30,31,41,42^. Our group reported the self-sorting of zinc-centered porphyrin monomers into homochiral stacks in solution and selective depolymerization of the chiral monomers by addition of a Lewis base^43^. We propose that the chiral discrimination exhibited in self-sorting systems could be used as a tool to isolate the contributions of interactions and effects, allowing to control the aggregate microstructure in porphyrin-based multi-component systems.

**Fig. 1:**
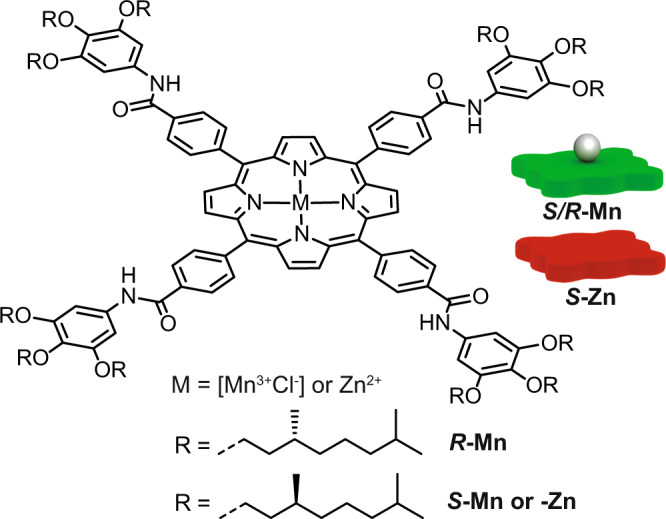
Molecular structure of *R*- or *S*-chiral porphyrin ligands. Inserted into the ligand is either a divalent zinc (***S*-Zn**) or trivalent manganese (***S***- or ***R***-**Mn**) ion. To balance the surplus charge on the Mn^3+^-ion the ligand has a chloride counterion bound to one side of the monomer.

In this study we focus on a zinc-centered porphyrin-based monomer (***S*-Zn**), which in apolar solvents such as methylcyclohexane (MCH) forms highly cooperative, helical supramolecular polymers through fourfold hydrogen bonding interactions. The ***S*-Zn** monomers exhibit pathway complexity and form next to helical H-aggregates also non-helical weakly coupled J-aggregates via an isodesmic mechanism^18,22,43,44^. We use monotopic manganese^III^ porphyrin monomers (***S/R*-Mn**) with an axially bound chloride ion as monotopic chain-cappers for zinc centered porphyrin monomers (Fig. 1)^45,46^. By adding homo- or heterochiral manganese chain-cappers we enantioselectively control the capping of H- or J-aggregates and reduce the length of the supramolecular polymers. Because of coupled equilibria, we can regain H-aggregates from depolymerized mixtures of manganese and zinc monomers by simply reducing the overall concentration of all components through dilution-induced supramolecular polymerization. With a combination of experiments and molecular modeling, we shed light on the complex interplay of interactions and how it is possible to exploit this knowledge to gain control over supramolecular aggregation in multi-component systems.

## Results

### Supramolecular homopolymerization

From previous studies the optical properties and polymerization mechanism of ***S*-Zn** in apolar solvents such as MCH are known^18,44^. Due to the difference in electronic coupling between monomers in the two (H- and J-type) aggregates, the H-aggregate exhibits an absorption band at λ = 392 nm with a strong bisignate cotton effect in circular dichroism spectroscopy (CD), while the J-type aggregate has its maximum absorption band at 425 nm and is CD silent (Supplementary Figs. 2, 3). Due to the axially bound chloride counterion, the monomers of ***S/R*-Mn** can only be monomeric or dimeric. In temperature-dependent melting experiments no spectral changes were measured at the maximum absorption wavelength of 475 nm, indicating that ***S/R*-Mn** is monomeric at dilute concentrations of *c* = 50 µM (Supplementary Fig. 2c, d). In diffusion ordered spectroscopy (DOSY) NMR experiments at c = 2.0 mM solutions in deuterated MCH we find short species with a diffusion coefficient of 2.32·10^−10^ m^2^·s^−1^ for ***S*-Mn**. This corresponds to a diameter of around 22.7 Å, which is in the range of dimers (Supplementary Fig. 8). Macroscopically, the formation of small monomeric or dimeric species for ***S*-Mn** is evidenced by excellent solubility and low viscosity in MCH as seen by tube inversion test of 2.0 mM solutions in MCH-*d* (Supplementary Fig. 5). For ***S*-Zn**, on the other hand, we only observe the diffusion of the solvent in DOSY-NMR experiments, as the long supramolecular fibers diffuse too slowly to be measured with this technique (Supplementary Fig. 9). Moreover, concentrated 2 mM solutions of ***S*-Zn** show high viscosity and gel-like properties due to the network formation of the supramolecular fibers (Supplementary Fig. 5). To further probe the ability of the two monomers to assemble, we conducted Fourier-transform (FT)-IR measurements of 2.0 mM solutions of ***S*-Zn** and ***S*-Mn** in MCH. The amide NH stretch vibration for ***S*-Mn** is found at 3321 cm^−1^ while for ***S*-Zn** this vibration is shifted to lower wavenumbers of 3289 cm^−1^, indicating hydrogen bonded supramolecular organization for ***S*-Zn** (Supplementary Fig. 6) and only weak hydrogen bonding for ***S*-Mn**. Notably, ***S*-Mn** shows two vibrations in the carbonyl region at 1650 and 1675 cm^−1^ in both bulk and solution spectra (Supplementary Figs. 6, 7). This hints to two different sets of carbonyl conformations in ***S*-Mn**, with one set as a free carbonyl in the monomeric state and the other being involved in the hydrogen bonding within the dimeric structures.

### Enantioselective chain-capping of porphyrin stacks

**Fig. 2:**
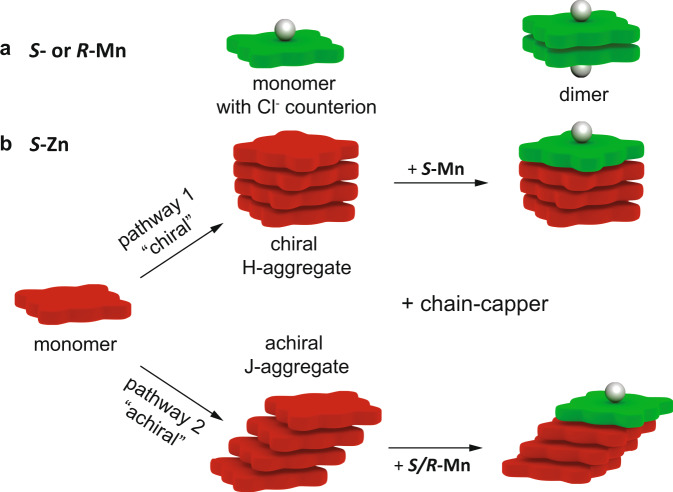
Aggregate types and pathways for a *S*-Mn and b *S-*Zn and co-assembled mixtures thereof. **a** While ***S/R*-Mn** can only be monomeric or form a dimer, (**b**) ***S*-Zn** can aggregate via a cooperative mechanism to H-aggregates or via an isodesmic pathway to J-type aggregates. The achiral J-aggregate can be chain-capped by both ***S-*** and ***R*-Mn**, but the chiral H-aggregate can only interact with ***S*-Mn** as a chain-capper, introducing the possibility for enantioselective chain-capping of polymers.

Porphyrin monomers are known to be highly narcissistic self-sorters, the energetic penalty for intercalating into an H-aggregated stack of the wrong helicity is high (~4 kJ/mol^−1^) so that forming both *M*- and *P*-stacks in solution is more favorable than copolymerization^43^. Therefore, only homochiral monomer/chain-capper pairs should lead to an efficient decrease in supramolecular chain length of the H-aggregates. The J-aggregates on the other hand are non-helical and their aggregation is mostly driven by π-stacking through solvophobic effects. The chirality of the monomer should have no influence in case of J-aggregates, hence, chain-capping can occur for both homo- and heterochiral mixtures (Fig. 2). When the two monomers ***S*-Zn** and ***S/R*-Mn** are mixed, there are several possibilities for co-aggregate formation: (1) no interaction and both monomers self-sort; (2) homochiral interaction, only ***S*-Mn** is a chain-capper for ***S*-Zn** polymers; (3) heterochiral interaction, both ***S****-* and ***R*-Mn** are chain-cappers for ***S*-Zn** polymers. Furthermore, the manganese centered monomers can act either as sequestrators or chain-cappers, the main difference being that the former interacts mostly with monomeric species and the later mostly with the chain-ends of H-aggregates. Both modes of action would reduce the chain-length, although with different efficiencies^47^.

**Fig. 3:**
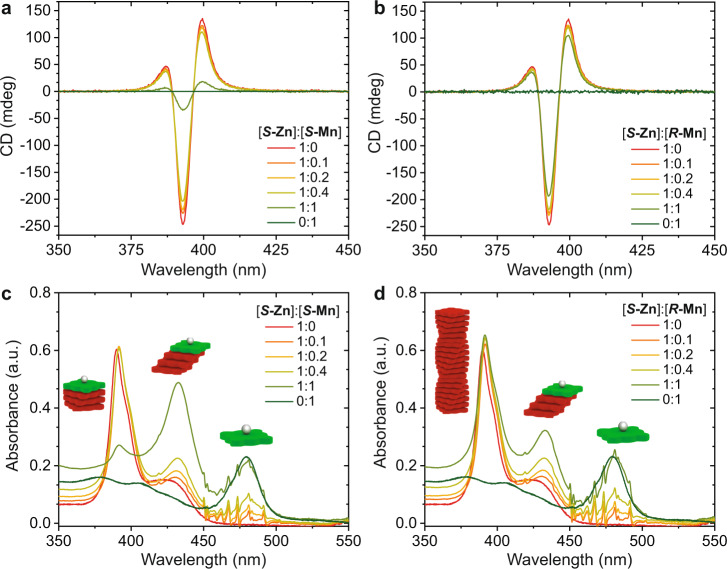
Enantioselective chain-capping in the mixed porphyrin system. **a** CD and (**c**) absorbance spectra of homochiral mixtures of ***S*-Zn** (c_***S*-Zn**_ = 25 μM in MCH) with increasing amounts of ***S*-Mn** chain-capper. With increasing molarity of ***S*-Mn**, the quantity of H-type aggregates at 392 nm decreases and more J-aggregates are formed (visible at 425 nm). **b** CD and (**d**) absorbance spectra of heterochiral mixture of ***S*-Zn** with ***R*-Mn**. Compared to the homochiral mixture far less H-aggregate is converted into J-aggregate as the monomers can only interact weakly via the isodesmic pathway with the achiral J-aggregate.

To test our hypothesis of enantioselective chain-capping, as displayed in Fig. 2, we prepared mixed solutions of ***S*-Zn** (*c*_***S*-Zn**_ = 25 μM in MCH) with either homochiral ***S*-Mn** or heterochiral ***R*-Mn**. To exclude dilution effects, we kept the concentration of ***S*-Zn** constant for all CD and absorbance measurements and added aliquots of the second component ***S/R*-Mn**. Each of the aggregate species has distinctive bands in the absorption spectra, allowing us to follow the transformation from H-aggregates (λ = 392 nm) to J-aggregates (λ = 425 nm) with increasing amounts of ***S*-Mn** (λ = 475 nm). For the homochiral monomer/chain-capper pair, a decrease in CD of the H-aggregate at 392 nm is observed when increasing the amounts of ***S*-Mn** to a 1:1 ratio at 25 μM in MCH (Fig. 3a). When the length of the H-aggregates decreases through the interaction with the chain-cappers, the H-aggregates become destabilized and convert into J-aggregates as the interaction energy of the aggregates decreases with decreasing length^3^. Similar to the CD spectra also the UV plots show an increase of J-aggregate at λ = 425 nm most strongly for the 1:1 mixture (Fig. 3a, c). For the heterochiral pair, however, these effects are much less pronounced (Fig. 3b, d). From 0 to 1 equivalent of ***R*-Mn** added, neither the CD spectra nor the absorbance spectra show a decrease of H-aggregates, but a slight increase of J-aggregates (Fig. 3b, d) as seen for the homochiral pair (Fig. 3a, c). With increasing J-aggregate formation the absorbance shifts to the visible range and the solutions change from green to a more reddish color for the homochiral, but not for the heterochiral capper/monomer pair (Supplementary Fig. 4). In the cartoons we only show one chain-capper per chain, however, double capping of the oligomers with ***S/R*-Mn** on both ends is also possible, but the experiments do not indicate one or the other. Here we have demonstrated that we can select the aggregation pathway of porphyrin supramolecular polymers through chiral discrimination with a “chiral” pathway for interacting with H-aggregates and an “achiral” pathway for sequestrating the monomers into J-aggregates (Fig. 2b). These results seem to support that only ***S****-***Mn** can act as a chain-capper for both the H- and J-aggregates of ***S*-Zn**, while ***R*-Mn** only interacts with the achiral J-aggregates and can probably sequester some monomers, but much less efficiently. To unravel the nature of interactions between ***S*-Zn** and ***S/R*-Mn** monomers, we continue with theoretical molecular dynamics calculations in the next section.

To gain insights into the length of the aggregates, we performed additional static light scattering experiments. The length of the aggregate can be determined from fitting the scattering plot with a cylindrical model^47–49^. However, even at concentrations as low as 10 µM in MCH, the supramolecular polymers formed by ***S*-Zn** are too long to be resolved, so that only qualitative information could be extracted (Supplementary Fig. 10). The scattering plots of homochiral mixtures of ***S*-Zn**/***S*-Mn** overlap except the curve of the 1:1 mixture, which shows the presence of a smaller aggregated species. All the mixtures of the heterochiral pair ***S*-Zn**/***R*-Mn** give a scattering profile indicative of long, cylindrical structures even at equimolar concentrations of monomer/chain-capper.

### Coarse-grained molecular modeling of chain-capping events

**Fig. 4:**
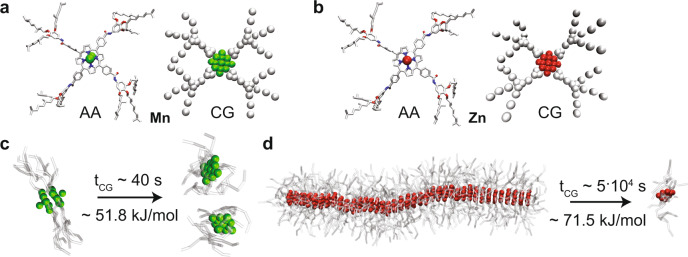
Molecular modeling. **a**, **b** All-atom (AA) and coarse-grained (CG) models for **Zn** and **Mn** monomers. The **Zn** fiber and the **Mn** dimer were equilibrated in cyclohexane. **c**, **d** The free energy barrier and the CG-time of exchange from the dimer and the fiber tip obtained from infrequent WT-MetaD simulations.

To help understanding the experimental findings, we developed a molecular model to describe the system. We initially built an all-atom (AA) model of the monomers (computational details in the Supplementary Information). However, the size and the timescales involved in the self-assembly and dynamic behavior of these supramolecular systems exceed the possibilities for AA models. As recently done for similar structures^14,50^, we thus developed coarse-grained (CG) models for the monomers which, based on the Martini CG force field^51^, have been further optimized to be consistent with the behavior of the AA models. The limited resolution of these CG models (3 heavy atoms per CG-bead) does not allow to distinguish between *S*- or *R*- chirality. Nonetheless, such CG model can safely be used to qualitatively compare the dynamics of two (**Mn** and **Zn**) porphyrin-based supramolecular building blocks, and to shed light on the complex mechanism of monomer exchange and of interaction between comonomers^15,52–54^. The model for **Zn** monomers was based on previous work on zinc centered porphyrin polymer fibers^14^. For the **Mn** monomers, we started from an AA model and we subsequently tuned the CG model in order to reproduce the porphyrin core–core dimerization free energy using a well-tested metadynamics (MetaD) protocol^14,15,50,52^. We used these CG models (Fig. 4a, b) to study and compare via MetaD simulations the **Zn-Zn**, **Mn-Mn** and **Zn-Mn** interactions^15,52^. Our MetaD simulations provided a dimerization ∆*G* for **Mn-Mn** cores of ~18.0 kJ/mol. In previous work, the ∆*G* for **Zn-Zn** was found a higher values of ~45.2 kJ/mol^14^. The **Zn-Mn** core interaction (~18.8 kJ/mol) on the other hand, was found just slightly stronger than **Mn-Mn** core interactions.

Subsequently, CG models for a **Mn** dimer and for a fiber composed of 40 **Zn** monomers were pre-equilibrated in a periodic simulation box filled with explicit cyclohexane solvent molecules (equivalent to MCH in our CG representation)^14^, and we performed molecular dynamics (MD) simulations at a temperature of 300 K and a pressure of 1 atm. Both the **Mn** dimer and the **Zn** fiber resulted in stable structures in the timescale accessible by these CG-MD runs (details on the simulations in the Supplementary Information).

We employed infrequent well-tempered metadynamics (WT-MetaD)^55^ simulations, using the same approach recently used for studying the dynamics of other supramolecular polymers, in order to obtain information on the relative characteristic kinetics for the events of **Mn** dimer dissociation and **Zn** monomer exchange from the **Zn** fiber tip^15,52,53^. Activating monomer exchange in the two systems, these simulations allowed us to retrieve the free energy barriers involved in the exchange and the related characteristic timescales (*t*_CG_) expected for these exchange phenomena. The **Mn** dimer breakage requires the system to cross an activation barrier of ~51.8 ± 0.8 kJ/mol. On the other hand, exchanging one monomer from the **Zn** fiber tip requires crossing a free-energy barrier of ~71.5 ± 2 kJ/mol. The transition probability distributions obtained from multiple infrequent WT-MetaD simulations (see Supplementary Fig. 11) allowed us to estimate the kinetics of monomer exchange and to compute the characteristic exchange timescale. While the exchange timescales estimated from these (approximated) CG models should be considered as qualitative, these are still useful to compare the dynamics of the two systems simulated at the same level of resolution^15,52–54^. The characteristic timescale for monomer exchange is found in the order of ~10^1^ s for the **Mn-Mn** dimer. In comparison, the **Zn** fiber appears as much more static. In this case, monomer exchange from the fiber tip occurs on a characteristic timescale of ~10^4^ s in agreement with the static nature of similar **Zn** porphyrin fibers reported recently^14^. These results indicate that exchanging a monomer with the solvent from a **Mn** dimer is ~1000 times faster (or more probable) than exchanging a monomer out from the **Zn** fiber tips (see Fig. 4). This makes finding free/disassembled **Mn** monomers in solution way more probable than finding disassembled ***Z*n** monomers. Moreover, once a **Mn** monomer is in solution, its binding onto a **Zn** fiber tip is likely, as the **Zn-Mn** interaction is similar (and slightly higher) than the **Mn-Mn** interaction. Therefore, the saturation of **Zn** fibers by binding of **Mn** monomers to the fiber tips is a likely event.

**Fig. 5:**
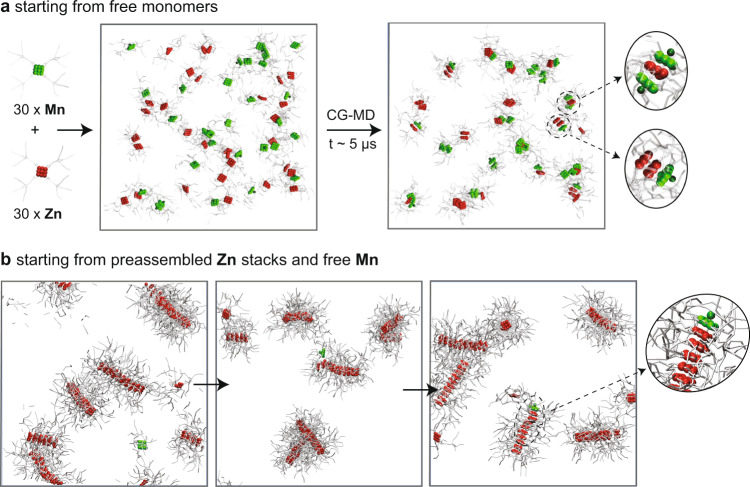
CG simulations of co-assembly of Mn and Zn monomers. Simulations starting from (**a**) free monomers or (**b**) preassembled **Zn** stacks and free **Mn**. All the simulations performed described above were carried out using GROMACS^60^ version 2018.6, patched with the PLUMED version 2.5.0^61,62^.

Next, we simulated two different model systems, (i) starting from free **Mn/Zn** monomers in solution that can freely interact and self-assemble (Fig. 5a) or (ii) starting from pre-formed **Zn** fibers surrounded by free/disassembled **Mn** monomers (Fig. 5b). We compare the behaviors of both systems by means of CG-MD simulations in explicit solvent. Based on the results on the exchange dynamics of the respective aggregate types, we would expect both sequestration and chain-capping to occur for the first case, while for the second case chain-capping should be most common as the **Zn** fibers exchange monomers very slowly. After 5 µs of CG-MD simulation time for the first scenario, short aggregated species are observed as spontaneously appearing in the system: these are homo- or hetero-dimers of the two monomers, sandwich type **Mn-Zn-Mn** complexes or short, chain-capped stacks of **Zn** (Fig. 5a). The occurrence of sandwich-type species in the simulations supports the previously mentioned probability that also longer stacks of ***S*-Zn** could be end-capped by two ***S/R*-Mn** monomers.

For the second case, since the spontaneous chain-capping event could not be observed within the timescale accessible via classical/unbiased CG-MD simulations, for explorative purpose we used WT-MetaD to accelerate the sampling of the system (a detailed description of the WT-MetaD setup is available in the Supplementary Information). This allowed us to qualitatively observe that **Zn** chain-capping by **Mn** is indeed possible in the system (Fig. 5b). Interestingly, we could observe that over the course of this simulation the **Mn** monomer first starts to interact with the surface of the **Zn** stack. After some time, it finally binds and chain-caps the preexisting supramolecular **Zn** fiber. Such a complex adsorbing-sliding-stacking mechanism is in line with what recently observed also for other types of supramolecular polymers^53,54,56^.

In order to obtain more robust evidence, we also conducted WT-MetaD simulations for the exchange of a single **Mn** monomer in a single **Zn**-fiber (see Supplementary Fig. 12a). From WT-MetaD, we could obtain a free energy surface for the **Mn** exchange process, which confirms such exchange pathway (Supplementary Fig. 12b). Kinetic analyses obtained from multiple infrequent WT-MetaD simulations (using the same approach used to calculate the exchange timescales of Fig. 4b, c) also demonstrate that, in this system, the interaction of **Mn** with the surface of a **Zn** fiber is relatively weak, while the directional interaction between the **Mn** and the **Zn** cores that is established upon **Mn** capping is considerably stronger (rate limiting step—see also Supplementary Fig. 12c). This confirms the **Mn** capping mechanism seen also in Fig. 5b, where a **Mn** monomer coming from the solution first interacts with the **Zn** fiber surface (i.e., the largest portion of the fiber, and the most probable to impact with), and then moves from this state until stacking onto the fiber tip (i.e., the most favorable bound state: see Supplementary Fig. 12 and the Supplementary Information for more details).

### Dilution-induced supramolecular polymerization

**Fig. 6:**
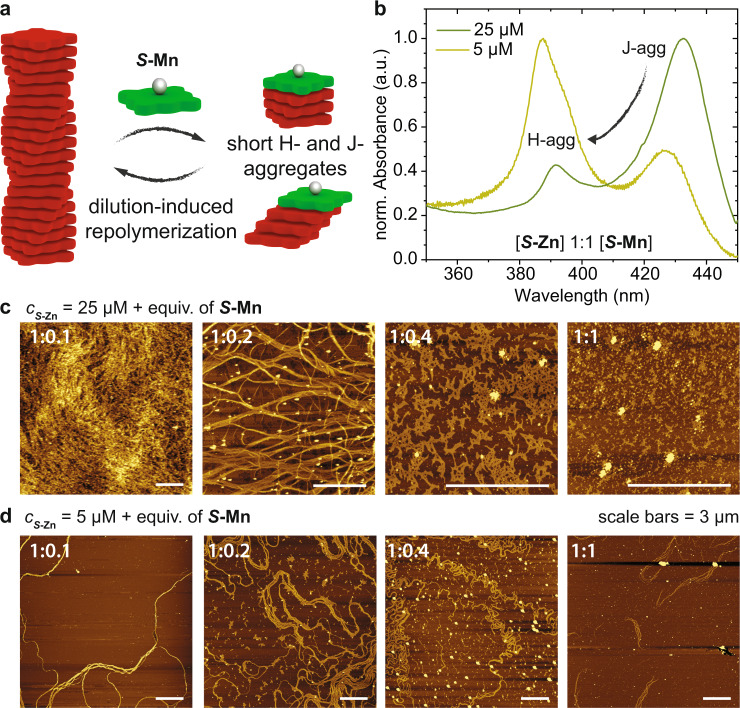
Dilution-induced supramolecular polymerization of porphyrin-based polymers. **a** Schematic representation of dilution-induced supramolecular polymerization process. **b** Absorbance spectra showing the dilution-induced repolymerization from J- to H-aggregates when decreasing the concentration from 25 to 5 μM for a 1:1 mixture of ***S*-Zn/*S*-Mn** in MCH. **c** AFM images at c_***S*-Zn**_ = 25 µM and (**d**) c_***S*-Zn**_ = 5 µM showing the depolymerization with added equivalents of chain-capper at high concentration, and the reforming of supramolecular fibers in dilute solutions. Samples were prepared by drop-casting the solutions on mica, scale bars represent 3 μm.

To visualize changes in aggregate morphology and length by the addition of ***S*-Mn**, we conducted a series of atomic force microscopy (AFM) experiments (Fig. 6c, d). At identical concentrations as in the spectroscopic experiments (*c*_***S*-Zn**_ = 25 µM), the surface is densely covered in long fibrous aggregates for ***S*-Zn** solutions but only small, non-fibrous deposits were observed for ***S*-Mn** (Supplementary Fig. 14). For mixtures thereof, a gradual transition from fibers to disordered material can be observed in the images (Fig. 6c), which supports that ***S*-Mn** causes the depolymerization and transformation of H- into J-aggregates of ***S*-Zn**. Intriguingly, when diluting the mixtures and repeating the AFM measurements at lower concentration (*c* = 5 µM, Fig. 6d), we witnessed the reformation of H-aggregates from the J-aggregates. At 5 µM, all samples show long fibrous structures with some exposed chain-ends. This is most apparent for the 1:1 mixture where we observed short bundles of fibers. For heterochiral mixtures of [***S*-Zn**]:[***R*-Mn**] at same concentrations, this effect is not as pronounced (Supplementary Fig. 15). At high molar ratios of ***R*-Mn** at 25 μM, the morphology found on the surface resembles the organization of J-aggregates on a surface as reported previously for a sequestered mixture of *N*- and *C*-centered porphyrins^3^. At 5 μM of ***S*-Zn** with 0 equivalents of chain-capper, we observe fibrous aggregates but without the exposed chain-ends as for the homochiral mixture. This counterintuitive repolymerization with decreased total concentration is a consequence of coupled equilibria and pathway complexity and has previously only been described in the dilution-induced self-assembly of ***S*-Zn** with a 40-fold excess of pyridine (Fig. 6a)^22^.

What is observed in the AFM experiments can also be seen in the CD and absorbance spectra when diluting from 25 to 5 µM (Fig. 6b and Supplementary Fig. 13). Solutions of ***S*-Zn**/***S*-Mn** at 25 µM show significant amounts of J-aggregates for the 1:1 mixture, which upon dilution to 5 µM transform into H-aggregates. In supramolecular polymerizations, dilution causes a decrease in chain length scaling with the square root of the concentration until ultimately below the critical aggregation concentration monomeric species form. Here, by lowering the concentration and shifting of the equilibria, the ***S*-Mn**/***S*-Zn** interaction becomes less favorable than ***S*-Zn** homo-interaction. This in turn causes a release of the ***S*-Mn** chain-capper from the chain ends and thereby reformation of the supramolecular polymers. For the dilution-induced supramolecular polymerizations the rate of sample equilibration was essential to avoid kinetic effects. Slow equilibration resulted in the formation of thermodynamically more stable H-aggregates, while for fast cooling rates kinetic trapping into J-aggregates was observed (Supplementary Fig. 16). So far, only two examples of this dilution-induced polymerization effect have been reported in supramolecular chemistry^22,57^. For proteins the refolding and renaturation by rapid dilution is a commonly used protocol, although the mechanism is different^58,59^. Here we reported a highly sensitive multi-component system, that is responsive to both dilution and the chirality of additives due to pathway complexity and coupled equilibria. By tailoring the specific interactions between ***S*-Zn** and homochiral additive ***S*-Mn**, we could significantly increase the sensitivity and effect on the chain-length compared to the ***S*-Zn**/pyridine system.

## Discussion

In this study we demonstrate how from coupled equilibria and complex pathways, intriguing and counterintuitive assembly behaviors can arise. We combine two monomers, a zinc centered porphyrin monomer (***S*-Zn**) which assembles into H-aggregates or J-aggregates, and a monotopic manganese porphyrin (***S/R*-Mn**) with an axially bound chloride ion that forms monomers or dimers and can act as a chain-capper for ***S*-Zn** stacks. We exploit the narcissistic self-sorting behavior of porphyrin monomers to enantioselectively discriminate between capping the helical H-aggregates or the achiral J-aggregates depending on the chirality of the chain-cappers. With increasing amounts of homochiral ***S*-Mn** chain-capper, both H- and J-aggregate of ***S*-Zn** get chain-capped. At equimolar ratios of [***S*-Zn**]:[***S*-Mn**], the H-aggregates become short and destabilized, resulting in conversion to J-aggregates. If ***R*-Mn**, a chain-capper of opposite chirality, is mixed with ***S*-Zn** this effect is much weaker as ***R*-Mn** can only interact with the achiral J-aggregates or monomers. Using coarse-grained molecular simulations, we can observe the underlaying dynamics of co-assembly and chain-capping events, revealing that multi-step processes (absorb-slide-stack) are likely involved in the exchange phenomena. AFM microscopy images show the transformation from long fibrous H-aggregates to short, disordered J-aggregates with increasing amount of homochiral chain-capper ***S*-Mn**. However, upon dilution this process can be reversed with the dilution-induced supramolecular polymerization effect. The two-component system of an either manganese and a zinc centered porphyrin ligand offers intriguing insights into the subtle rules and balances governing the behavior of supramolecular systems. Gaining control over aggregate morphology and length and aggregation pathways remains the dream of supramolecular chemists. Here we demonstrate that we can select the aggregation pathway and modulate the length by choosing either a homo- or heterochiral additive, and by decreasing the concentration with dilution-induced supramolecular polymerization. Understanding these complex assembly phenomena in supramolecular systems is paramount for moving towards functional, multi-component systems in the future.

## Methods

### Sample preparation

Stock solutions (100 μM) were prepared by weighing the compound and transferring it in a volumetric flask. One milliliter of a polar solvent (dichloromethane or chloroform) was added to break up aggregates and re-evaporated. Then the flask was filled up to the meniscus with methyl cyclohexane (MCH), heated and sonicated until complete dissolution. Samples for measurements were prepared by diluting the stock solution to the respective concentration. Before measuring mixed samples of Mn- and Zn-porphyrins the samples were thermally equilibrated by heating the sample solutions in a fitted metal block to 90 °C and slowly cooling to room temperature (cooling ramp  ~ 15 °C/h). The solutions were immediately measured the next day.

### Spectroscopy

UV/Vis and circular dichroism (CD) spectra were recorded on a Jasco J-815 spectropolarimeter with a Jasco PFD-425S/15 Peltier. The measurement parameters for sensitivity, scanning rates and ranges were chosen appropriately for each experiment. Cuvette cells with an optical path length of 1 mm were used for all experiments.

### Atomic force microscopy

The sample solutions used in the spectroscopic experiments were also used for the AFM experiments by dropcasting 20 µL of solution onto a 1 × 1 cm mica sheet (V1 quality). After complete evaporation of the solvent, the samples were measured on an Asylum MFP3 system with soft NanoWorld NCSTR50 tips in non-contact tapping mode.

### Molecular modeling and simulations

All simulations have been performed with GROMACS 2018.6^60^, patched with the PLUMED plugin 2.5.0^61,62^. The CG model for the **Zn** porphyrin monomer was adapted from our previous work on a similar system^14^. The AA and CG models for the **Mn** porphyrin monomer were developed, based on the GAFF^63^ and the MARTINI force fields^51^, respectively, following the same procedure^14^ (see Supplementary Information for complete details).

The free energy surface (Supplementary Fig. 12b) was obtained from a well-converged WT-MetaD^64,65^ simulation, using the distance between the central **Mn** bead and the central **Zn** bead of the closest neighbor monomer in the Zn-fiber as the CV1, and the number of contacts between the core of the **Mn** monomer and the core of all the **Zn** fiber as CV2. During the WT-MetaD simulation, the system successfully diffuses in the entire CV space, recrossing between the visited relevant states, reaching a satisfactory convergence.

The kinetics of the monomer exchange events have been estimated following a validated procedure^53^, by running multiple infrequent WT-MetaD^55^ simulations. The collected transition times were proven to fit well the typical Poisson distribution expected for a rare exchange event, validating the adopted setup^53,66^. From the fits, we estimated the characteristic transition times expected for monomer exchange in the various cases^15,52,53^.

## Supplementary information

Supplementary information

## Data Availability

The authors declare that the experimental data supporting the findings of this study are available within the paper and its Supplementary Information file. Complete details of the procedures for the parametrization of the molecular models and of the simulations’ setup, along with additional simulation data, are provided in the Supplementary Information file. Complete data and materials pertaining to the molecular simulations conducted herein (input, model files, raw data, simulation trajectories, etc.) are available at: 10.5281/zenodo.5717968. All other information is available from the corresponding author upon reasonable request.
