# Supplementary material for: Controlling the length of porphyrin supramolecular polymers via coupled equilibria and dilution-induced supramolecular polymerization
Source: Nat Commun. 2022 Jan 11;13:248. doi: 10.1038/s41467-021-27831-2 (PMC8752679; doi:10.1038/s41467-021-27831-2)
Supplement: Supplementary file 1 — Supplementary information [file 41467_2021_27831_MOESM1_ESM.pdf]

Supplementary Information

Controlling the length of porphyrin supramolecular polymers via coupled equilibria and dilution-induced supramolecular polymerization

Elisabeth Weyandt,[1,2] Luigi Leanza,[3] Riccardo Capelli,[3] Giovanni M. Pavan,[3,4] Ghislaine Vantomme,[1,2] and E.W. Meijer[1,2*]

[1] Laboratory of Macromolecular and Organic Chemistry, Eindhoven University of Technology, P.O. Box 513, 5600 MB Eindhoven, The Netherlands

[2] Institute for Complex Molecular Systems (ICMS), Eindhoven University of Technology, P.O. Box 513, 5600 MB Eindhoven, The Netherlands

[3] Department of Applied Science and Technology, Politecnico di Torino, 10129 Torino, Italy

[4] Department of Innovative Technologies, University of Applied Sciences and Arts of Southern Switzerland, 6962 Lugano-Viganello, Switzerland

* to whom correspondence should be addressed (e.w.meijer@tue.nl)

Table of Contents

1. Supplementary Methods: 3

2. Supplementary Discussion 3

2.1 Synthesis 3

2.2 Self-assembly in apolar solvents 4

2.3 All-atom and coarse-grained modeling of monomers 9

2.4 Dilution-induced supramolecular polymerization 14

2.5 Spectroscopic data of compounds 17

3. Supplementary References 18

1. Supplementary Methods:

Materials

Unless stated otherwise, reagents and solvents were obtained from commercial sources and used without further purification. All solvents were of AR grade or of spectrophotometric grade, when used in spectroscopy experiments. Reactions were conducted under argon atmosphere and dried glassware unless noted otherwise. Flash column chromatography was done on a Biotage Isolera One system with an ultraviolet detector.

Characterization

DOSY NMR measurements were conducted on a Bruker 400 MHz Spectrometer in deuterated methyl cyclohexane (MCH-*d*). MALDI-TOF mass spectra were measured using a PerSerptive Biosystem Voyager-DE PRO spectrometer using either α-cyano-4-hydroxycinnamic acid (CHCA) or 2-[(2E)-3-(4-tert-butylphenyl)-2-methylprop-2-enylidene]malononitrile (DCTB) as matrices. IR spectra were obtained on a Perkin-Elmer spectrum two FT-IR spectrometer. Static light scattering experiments were done on an ALV Compact Goniometer System (CGS-3) with a Multidetector (MD-4), an ALV-7004 Digital Multiple Tau Real Time Correlator. The wavelength of the laser was 532 nm. Scattering intensities were measured at scattering angles (ϑ) from 30 to 150˚ in steps of 10˚ averaged over 50 runs of 2 seconds per angle. Samples were filtered using Anopore® syringe filters (0.1 µm pore size).

2. Supplementary Discussion

2.1 Synthesis

The synthesis of free base ligand ***S*-FB** and ***R*-FB** and zinc porphyrin ***S*-Zn** has been published previously.[1,2]

Synthesis of manganese porphyrins *S*-Mn and *R*-Mn[3]

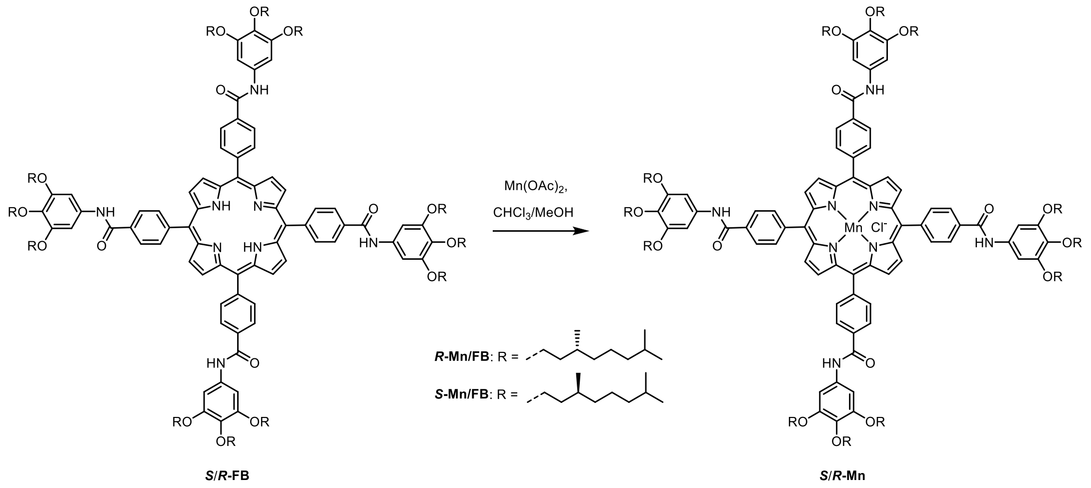

Supplementary Figure 1: Synthesis of *S/R*-Mn through Mn-insertion.

Ligand ***S*-FB** or ***R*-FB** (500 mg, 0.17 mmol) was suspended together with anhydrous $Mn(OAc)_2$ (500 mg, 2.89 mmol) in 20 mL chloroform with 5 mL methanol and refluxed overnight with protection from light. The green reaction mixture was extracted with 3x20 mL of 1M HCl, dried over $MgSO_4$, filtered and the

solvent removed in vacuo. The product was purified by column chromatography (Biotage®, gradient of ethyl acetate/chloroform) and isolated as a green powder (yield: 65-70%).

S-Mn:

The paramagnetism of ***S*-Mn** precludes NMR characterization. MALDI-TOF MS: [M^+] calcd. for $C_{192}H_{288}N_8O_{16}Mn$: 3017.13, found 3019.05. IR (cm^{-1}): 2953, 2926, 2869, 2717, 1702, 1604, 1592, 1453, 1382, 1366, 1349, 1316, 1296, 1248, 1166, 1056, 946, 845, 726, 714, 599.

R-Mn:

The paramagnetism of ***R*-Mn** precludes NMR characterization. MALDI-TOF MS: [M^+] calcd. for $C_{192}H_{288}N_8O_{16}Mn$: 3017.13, found 3019.22. IR (cm^{-1}): 3329, 2953, 2925, 2869, 1675, 1651, 1603, 1532, 1504, 1467, 1423, 1383, 1366, 1340, 1302, 1229, 1209, 1169, 1108, 1009, 900, 885, 824, 802, 760, 715, 667, 624.

2.2 Self-assembly in apolar solvents

Homopolymerization of *S*-Zn and *S*-Mn

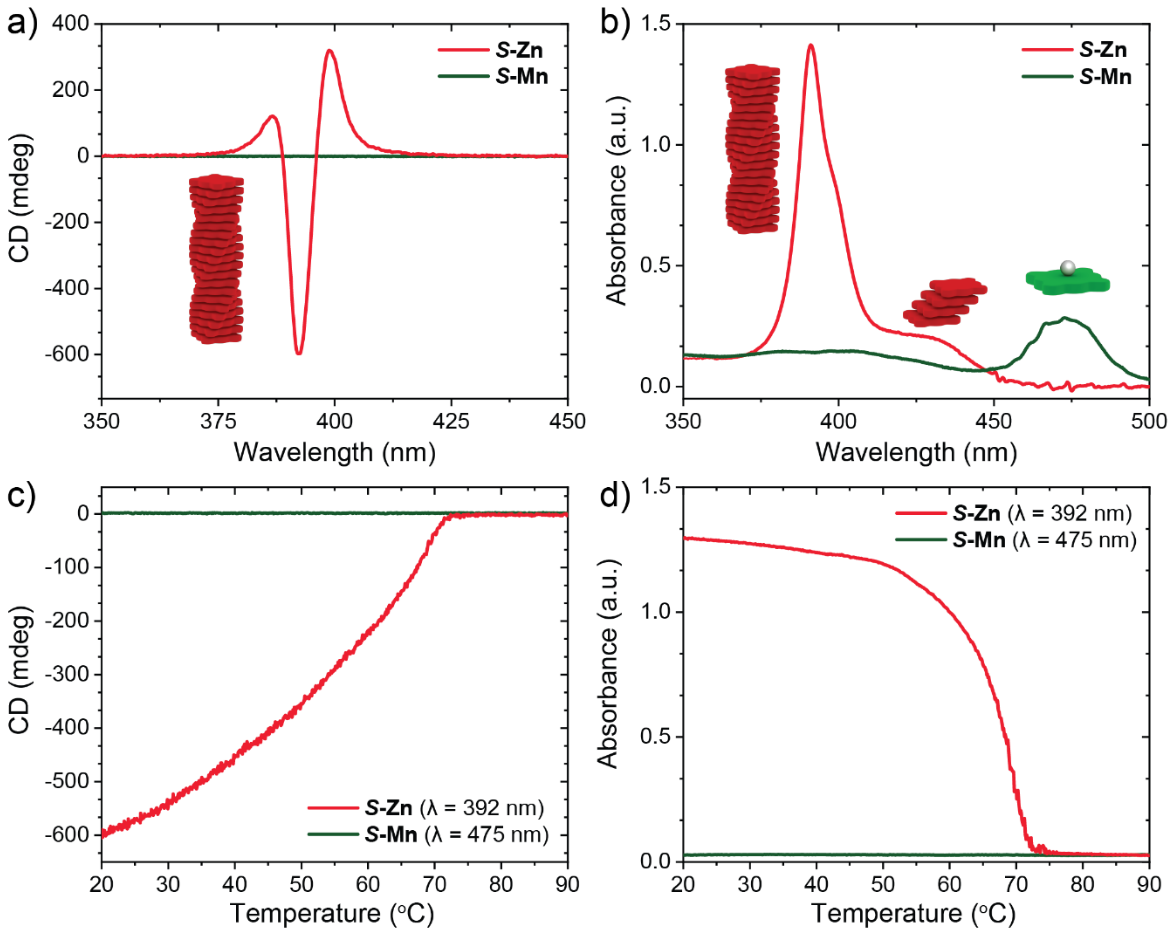

Supplementary Figure 2: Homopolymerization of ***S*-Zn** and ***S*-Mn** (*c* = 50 µM in MCH, pathlength *l* = 1mm). Full CD (a) and absorbance (b) spectra of the monomers, showing the presence of helical H-aggregate at 392 nm for ***S*-Zn** and small quantities of J-aggregates at 425 nm. The absorbance maximum for ***S*-Mn** is found at 475 nm. CD (c) and absorbance (d) melting curves show the cooperative polymerization of the H-aggregate for ***S*-Zn** at 392 nm and no temperature-dependent changes for ***S*-Mn** at 475 nm.

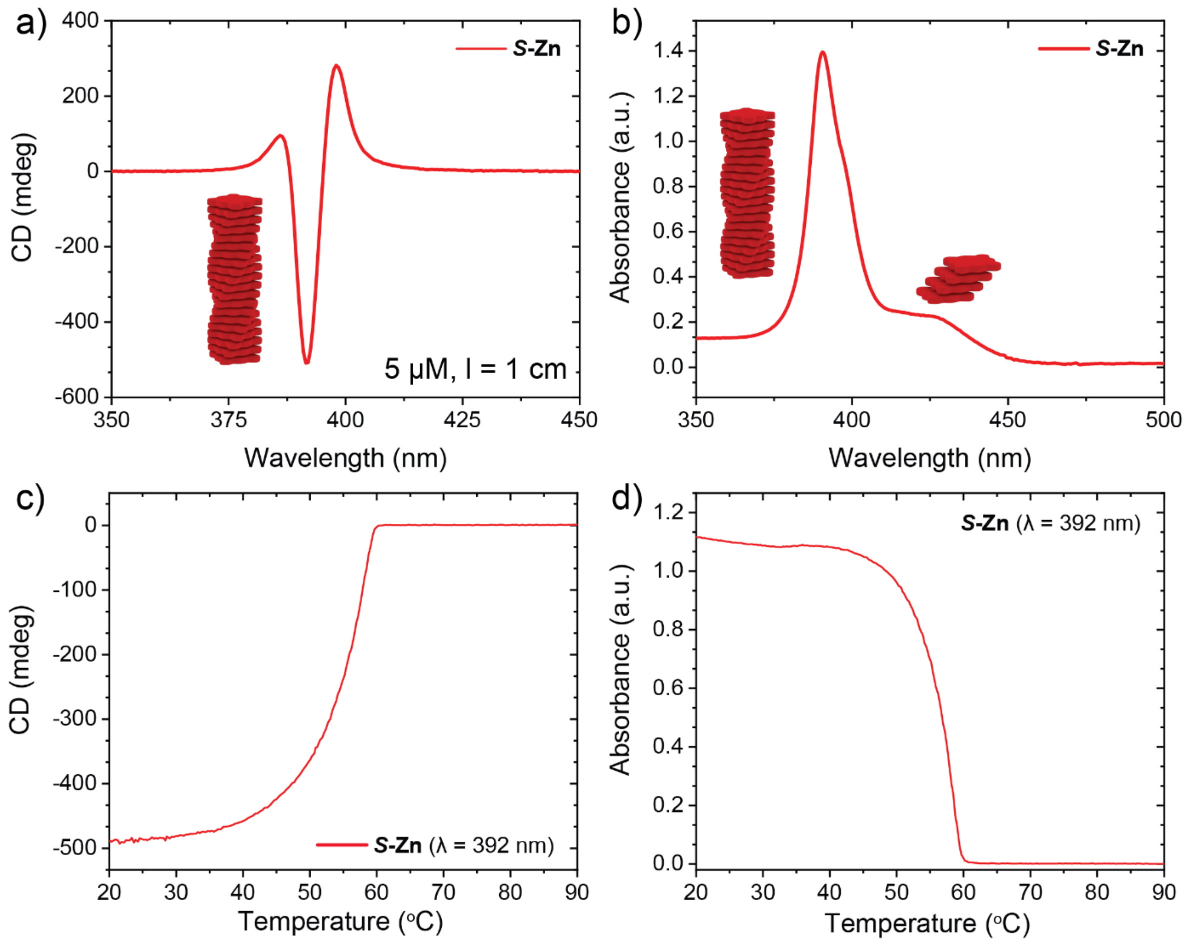

Supplementary Figure 3: Homopolymerization of ***S*-Zn** (c = 5 µM in MCH, pathlength l = 10 mm). Full CD (a) and absorbance (b) spectra of the monomer, showing the presence of helical H-aggregate at 392 nm for ***S*-Zn** and small quantities of J-aggregates at 425 nm. CD (c) and absorbance (d) melting curves show the cooperative polymerization of the H-aggregate for ***S*-Zn** at 392 nm.

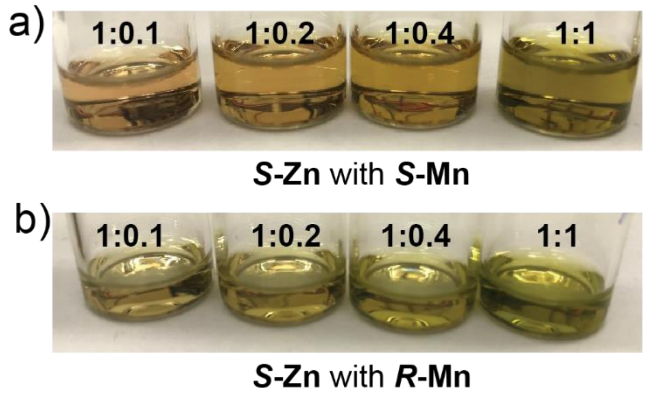

Supplementary Figure 4: Images of vials containing mixtures of [*S*-Zn]:[*S*-Mn] or [*S*-Zn]:[*R*-Mn] showing a more red-shifted color for homochiral mixtures than for heterochiral mixtures.

Gelation of *S*-Zn and *S*-Mn

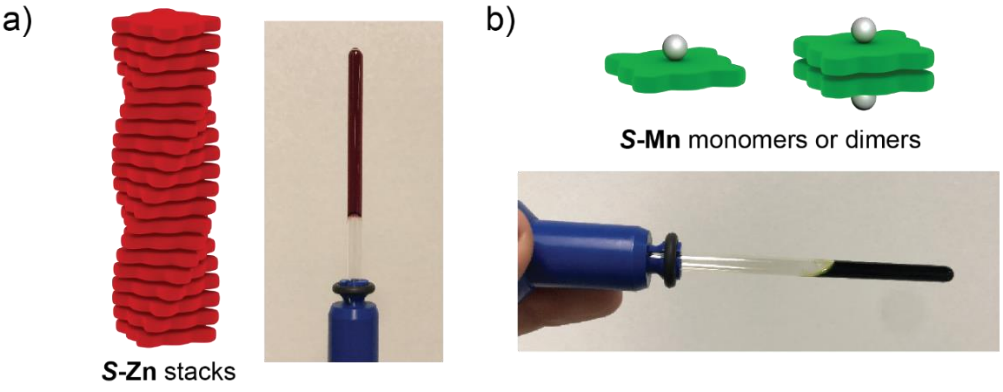

Supplementary Figure 5: Solutions of ***S*-Zn** in MCH-*d* (2.0 mM) show increased viscosity due to the formation of a fibrous gel network. a) Upon NMR tube inversion, the gel remains in the tip of the tube. b) A solution of ***S*-Mn** (2.0 mM in MCH-*d*) does not withstand the inversion test.

Fourier-transform infrared spectroscopy

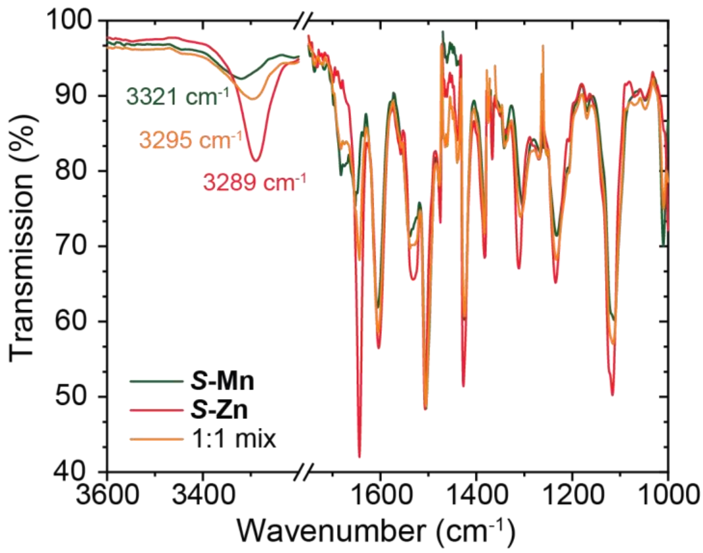

Supplementary Figure 6: Solution FT-IR spectra of ***S*-Mn**, ***S*-Zn** and a 1:1 mixture (*c* = 2.0 mM in MCH).

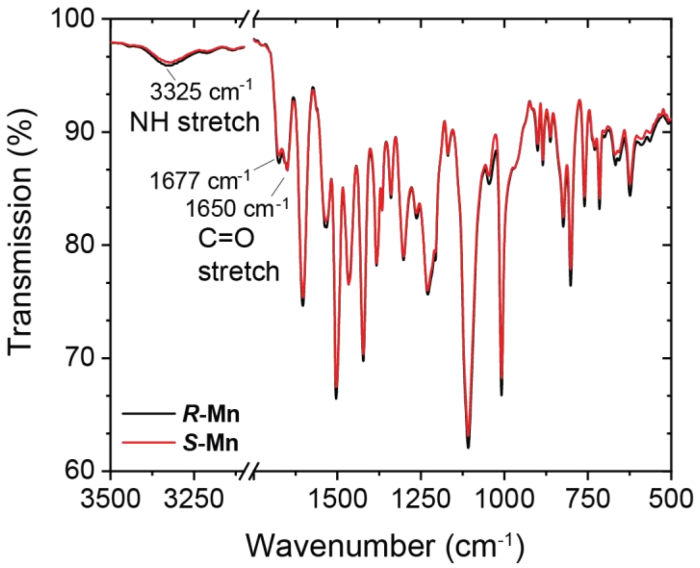

Supplementary Figure 7: FT-IR spectra of solid *S*-Mn and *R*-Mn with the NH and C=O stretch indicated in graph.

Diffusion ordered spectroscopy (DOSY) NMR

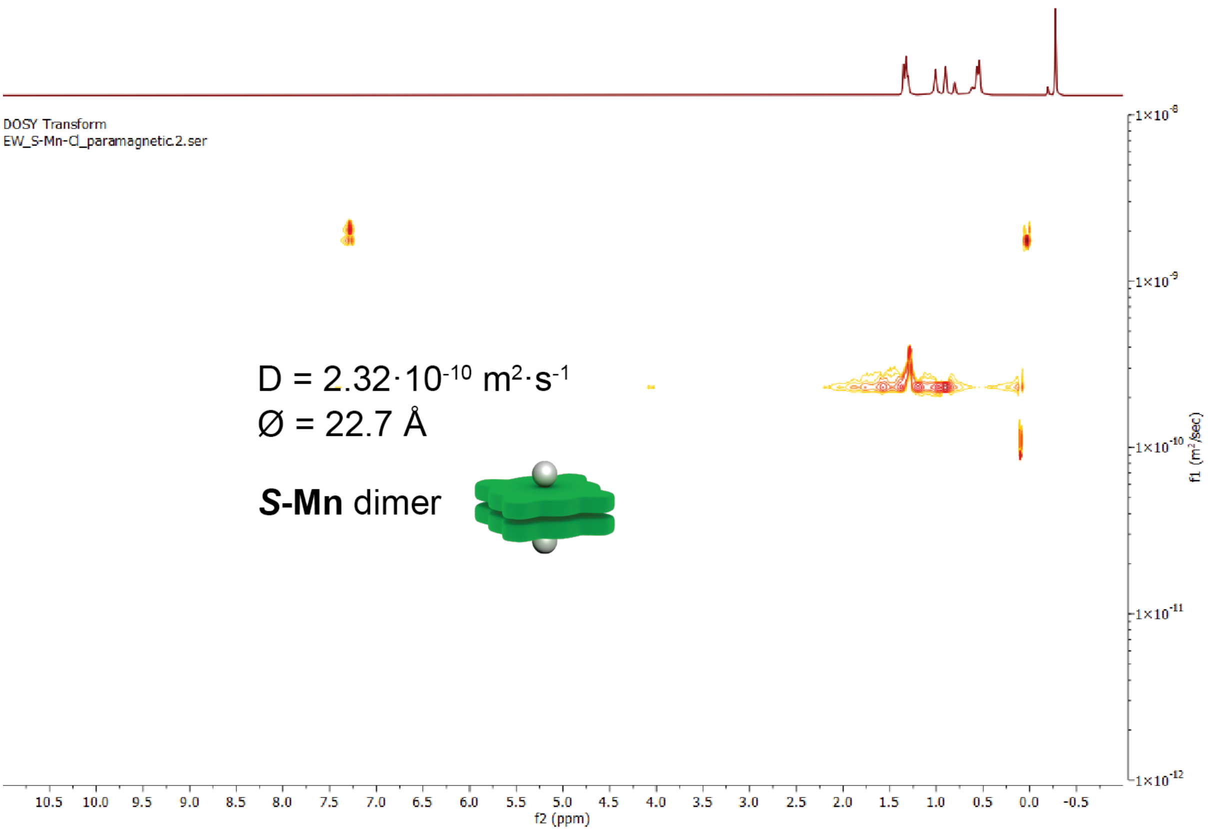

Supplementary Figure 8: DOSY NMR of 2.0 mM solution of *S*-Mn in MCH-*d*.

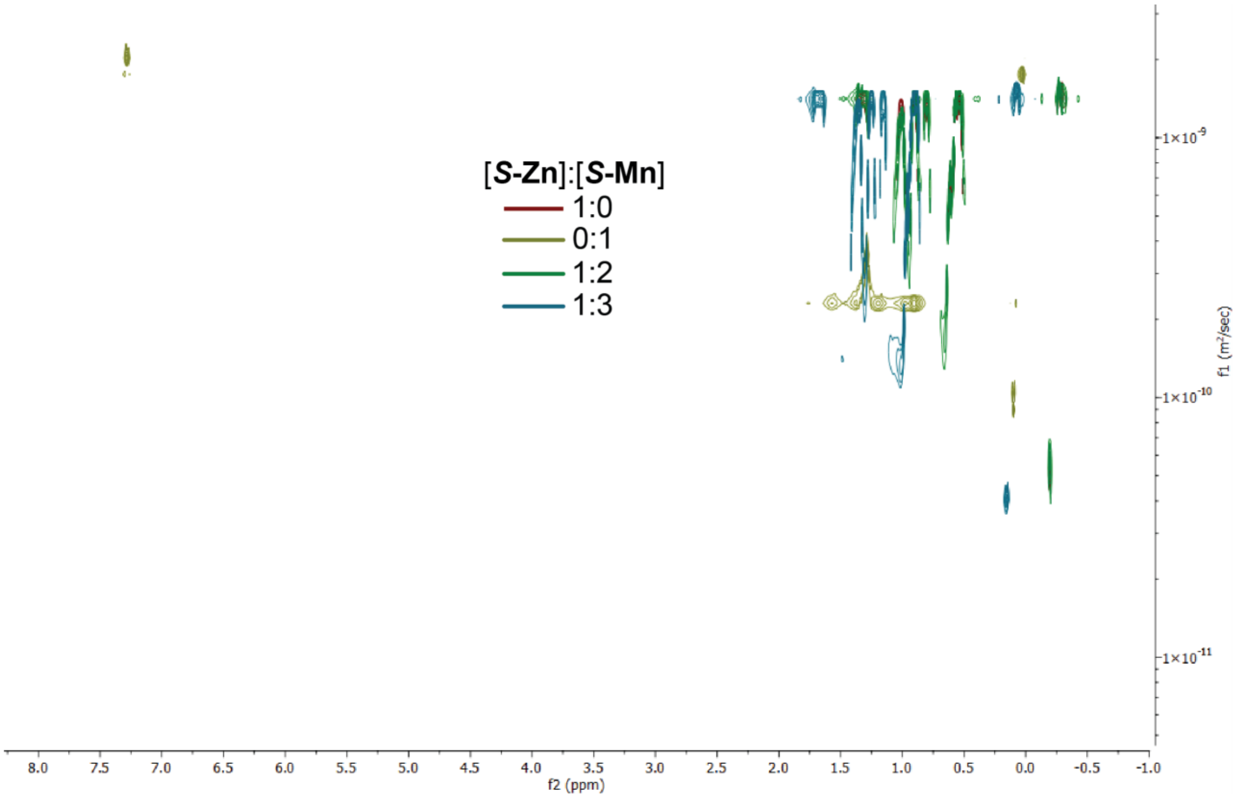

Supplementary Figure 9: DOSY NMR of 2.0 mM solution of *S*-Zn and 1:2 or 1:3 mixtures with *S*-Mn.

Static light scattering of porphyrin solutions

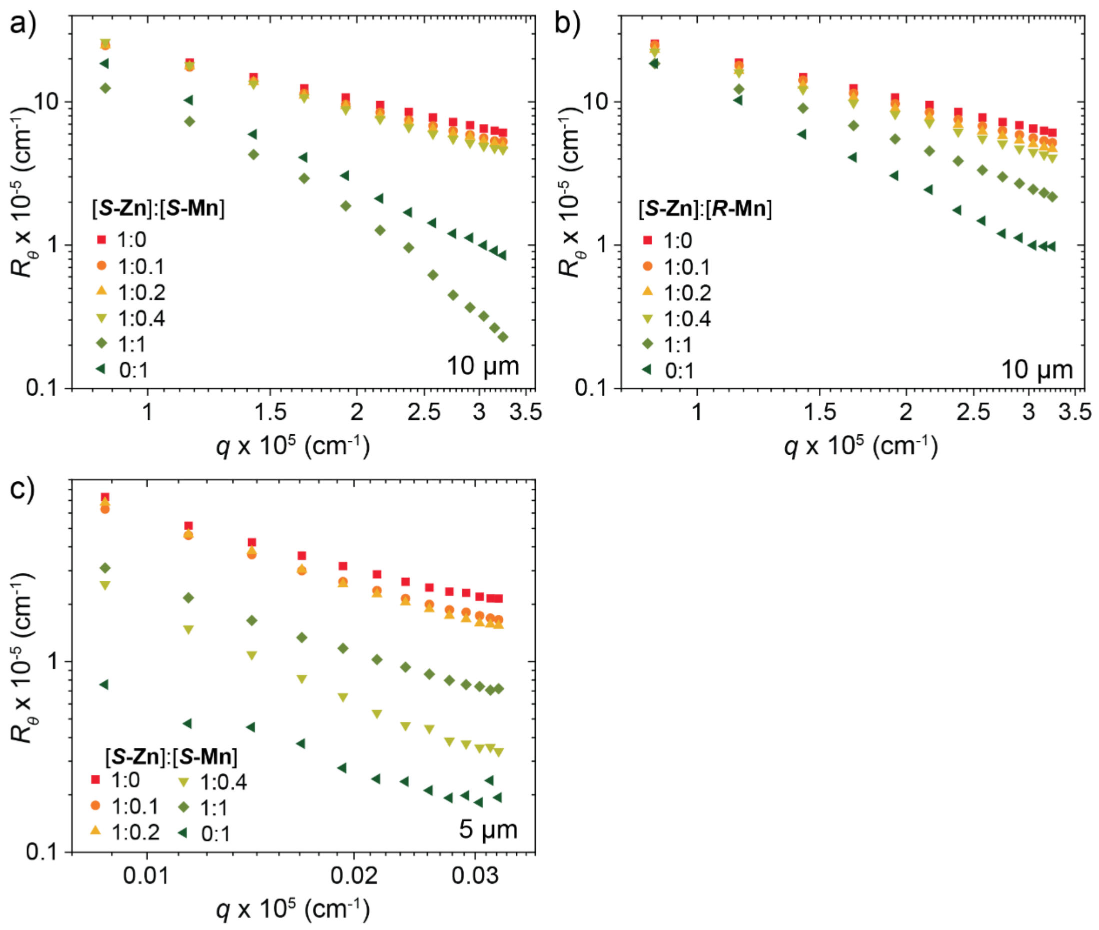

Supplementary Figure 10: Static light scattering profiles of mixtures of ***S*-Zn** (c = 10 μM) plus varying ratios of a) ***S*-** or b) ***R*-Mn.** At 10 μM the length of the ***S*-Zn** stacks is still too long to be fitted as can be seen from the almost linear increase in R_Θ over the range of q measured. Only for homo-chiral pair of 1:1 ***S*-Zn** with ***S*-Mn** the shape of the profile approaches a plateau at low q values, indicating shorter species. c) Scattering profiles of ***S*-Zn** (c = 5 μM) plus varying ratios of ***S*-Mn.**

2.3 All-atom and coarse-grained modeling of monomers

Development of the CG molecular models

The coarse-grained (CG) model of **Zn** monomer core used here has been already published in a previous work.[4] The all-atom (AA) model of **Mn** monomer was built using General Amber Force Field (GAFF)[5] with RESP[6] charges computed at B3LYP level of theory with LANL2DZ/6-31+G** mixed basis set and used as a reference to tune the CG model of **Mn** porphyrin, based on the MARTINI force field mapping.[7] Due to the complexity of the systems and the fact that arms are identical between **Mn** and **Zn**, we focused on the study of the porphyrin cores, similarly to what has previously done in the literature with supramolecular polymers modelling.[8–10] Two **Mn** porphyrin cores and a **Mn-Zn** dimer have been placed in two different pre-equilibrated simulation boxes of cyclohexane (CHX) and their dimerization free energy have been estimated via well-tempered metadynamics (WT-MetaD)[11]. The **Mn-Mn** interaction in solvent was found ~18 kJ/mol, slightly lower than the interaction between **Zn** and **Mn** (~18.8 kJ/mol) and significantly lower than **Zn-Zn** (~45.2 kJ/mol, obtained from the literature).[4] Then, the non-bonded

parameters of the **Mn** model were tuned in order to have a good agreement with the **Mn-Mn** dimerization energy obtained with AA simulations. Complete structures and parameters of the AA and CG models for the **Zn** and **Mn** monomers used herein are available at: https://zenodo.org/record/5717968.

Molecular simulations

All the simulations have been performed with GROMACS[12] version 2018.6, patched with the PLUMED[13,14] plugin version 2.5.0 and conducted at 300K. In AA production runs we used leap-frog integrator with a time step of 2 fs, the v-rescale thermostat[12] with a coupling constant of 0.2 ps and Parrinello-Rahman barostat[15] with a coupling constant of 4 ps. Non-bonded interactions were treated with a cutoff distance of 0.9 nm. Long-range electrostatic interactions were evaluated using the Particle Mesh Ewald (PME) method.[16] In CG production runs we used the leap-frog integrator with a time step of 10 fs, the v-rescale thermostat[17] with a time constant of 1 ps and Berendsen barostat[18] with a time constant of 4 ps. All the AA and CG WT-MetaD simulations for the estimation of dimerization free energies have been performed using the distance between metal atoms of the two monomers as collective variable. We used a bias factor of 25, a gaussian height of 0.4 kJ/mol and a deposition rate of 1 gaussian every ps and 5 ps in AA and CG, respectively.

Modeling monomer exchange from fiber tips and dimerization

The exchange of monomers out from an assembly (fiber or dimer) is a rare event at the timescale accessible to classical AA and CG simulations. We thus employed infrequent WT-MetaD[19] CG simulations activating the monomer exchange in the two systems. It is worth mentioning that the collected exchange timescales are calculated from simplified CG models and it is known that dynamics in CG simulations is accelerated and the conversion in real time is not straightforward. Nevertheless, the results are still useful to have a relative comparison between different models with the same CG level.

First, we built a **Mn** dimer and a fiber composed of 40 **Zn** monomers and equilibrated them in CHX. To obtain a transition probability distribution, we run 20 infrequent WT-MetaD simulations. The calculated residence times (t) were used to build a transition probability distribution $P_{n\geq1}$ in Supplementary Equation 1:

$$P_{n\geq1} = 1 - e^{-\frac{t}{\tau}} \qquad \text{Supplementary Equation 1}$$

The transition probability distributions fit well with a Poisson distribution, proving that we are measuring a rare event[20] (Supplementary Figure 11). We observe that the characteristic CG timescale of monomer exchange for **Mn** dimer is τ~40 s and it requires crossing a free energy barrier of ~51.9 ± 0.8 kJ/mol. The **Zn** fiber appears more static, with a τ~10^4 s and an activation energy of ~71.5 ± 2.1 kJ/mol. This calculation shows that exchanging a monomer from the **Mn** dimer is ~1000 times faster than exchanging a monomer from the **Zn**-stack. All the simulations were performed using two collective variables (CVs):

1) **Mn** dimer: the distance between the central beads of manganese (CV1) and the number of contacts between the two monomers (CV2).

2) **Zn** fiber: the distance between the central beads of zinc (CV1) and the number of contacts between the last monomer and the rest of the fiber (CV2).

The number of contacts $\mathrm{s_{ij}}$ was calculated using the following switching function in Supplementary Equation 2:

$$s_{ij} = \frac{1-\left(\frac{r_{ij}-d_0}{r_0}\right)^n}{1-\left(\frac{r_{ij}-d_0}{r_0}\right)^m} \qquad \text{Supplementary Equation 2}$$

where $\mathrm{r_0}$=0.3 nm, n=6, m=12, $\mathrm{d_0}$=0.

We used a bias factor of 10, a gaussian height of 1 kJ/mol and a deposition rate of 1 gaussian every 200 ps. The simulation parameters are identical to those of sect. 4.2.

Self-assembly and chain-capping simulations

We performed two CG-MD simulations starting from different initial conformations, namely: 1) 30 **Mn** and 30 **Zn** free monomers, and 2) one free **Mn** monomer and six pre-stacked **Zn**-fibers consisting of 10 monomers. The first system was simulated for 5 µs. During the simulation, we observed the formation of short homo- or hetero-dimers, sandwich type **Mn-Zn-Mn** complexes or short, chain-capped stacks of **Zn** (see Figure 5 in the main text). In the second simulation, the spontaneous chain-capping event is not observable from the timescale explored by unbiased CG-MD simulations, thus we sped up the process employing WT-MetaD. This simulation was performed using two CVs: 1) the minimum distance between **Mn** monomer and **Zn** monomers at the tip of the fibers and the 2) minimum distance between **Mn** monomer and all the **Zn** monomers of the fibers. We used a bias factor of 50, a gaussian height of 1 kJ/mol and a deposition rate of 1 gaussian every 5 ps.

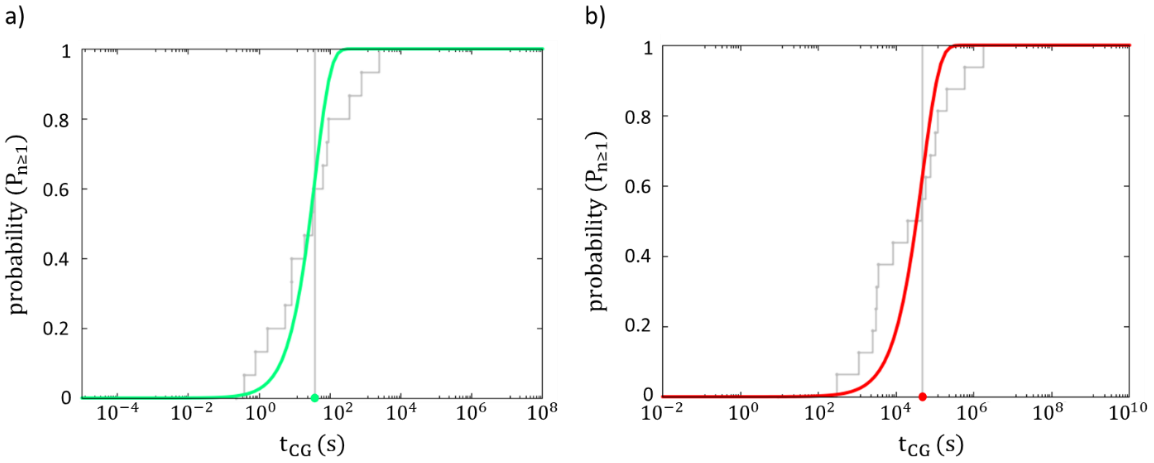

Supplementary Figure 11: Transition (monomer exchange) times estimated from multiple infrequent WT-MetaD simulations (each transition time is identified by the vertical grey segments) and Poissonian probability distributions for the detachment of one **Mn** monomer from a **Mn** dimer (a: in green) and of a **Zn** monomer out from a **Zn** fiber (b: in red). From the Poissonian fits, it was then possible to estimate the characteristic timescales for the monomer exchange events (τ) as defined above (in section 4.3), identified in these plots by the colored dots.

Elucidating the monomer exchange pathways

To clarify the interaction mechanism between **Mn**-based chain capper and the **Zn**-based fiber, we performed a CG WT-MetaD simulation. A **Mn** monomer was placed on the tip of a fiber formed by 30 **Zn** monomers, and pre-equilibrated. Two CVs were used in this CG WT-MetaD simulation: the distance between the central **Mn** bead and the central **Zn** bead of the closest neighbor monomer in the Zn-fiber (CV1), and the number of contacts between the core of the **Mn** monomer and the core of all the **Zn** fiber (CV2). We used a bias factor of 25, a gaussian height of 1 kJ/mol and a deposition rate of 1 gaussian every 5 ps. Given the large conformational space that can be explored during the capping/decapping events, and the size of the system, we used a 1.5 nm cutoff for CV1. During the simulation, we observe the **Mn** monomer binding and unbinding to the Zn-fiber multiple times. This approach allowed us to obtain insight into the most probable exchange pathways in the system (Supplementary Figure 12a).

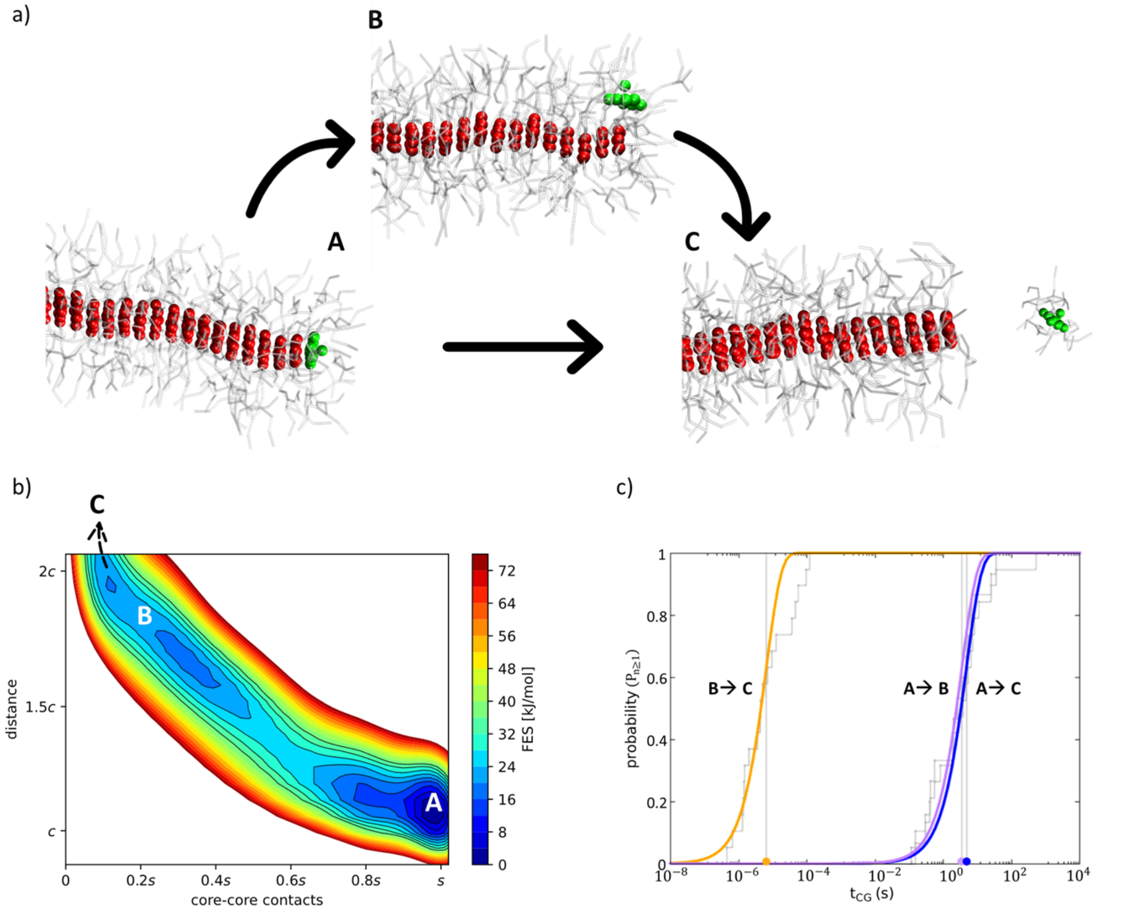

Supplementary Figure 12: Mechanisms of **Mn**-monomer exchange in/out a **Zn**-fiber. (a) WT-MetaD simulations biasing the unbinding/binding of a green **Mn**-monomer from/onto the tip of a **Zn**-fiber allow to study the possible pathways for monomer exchange (*e.g.*, A->B + B->C: stacked to adsorbed + adsorbed to solution, and back *vs.* A->C: stacked to solution in a single step). (b) Free energy surface (FES) obtained from WT-MetaD: the dark colors identify the most favorable exchange pathway. (c) Transition times distributions (gray vertical segments) and related Poissonian fits (colored curves) allowing to estimate the characteristic timescales (colored dots on the *x* axis) for the exchange transitions A->B (violet), B->C (orange), and A->C (blue).

From this CG WT-MetaD simulation trajectory, we computed the free energy surface (FES) of the **Mn**-monomer exchange as a function of CV1 and CV2 (Supplementary Figure 12b). The FES shows a global

minimum (Supplementary Figure 12b: A, dark-blue) at **Mn**-**Zn** stacking distance *c* (∼0.45 nm in this CG model), and number contacts between the **Mn**-**Zn** cores *s* (∼5 bead-bead core contacts in this CG model). State A identifies a system configuration where the green **Mn**-monomer is stacked onto the (red) **Zn**-fiber tip. A second broader local minimum (B) is found at larger **Mn**-**Zn** core-core distance and reduced number of contacts between the **Mn**-**Zn** cores (∼10-40% of the stacked state A). State B identifies the configurations where monomer **Mn** is de-stacked from the fiber tip, and adsorbed on the arms of the **Zn**-fiber (Supplementary Figure 12a; B). From state B, the **Mn**-monomer can then jump in solution (C).

We then used multiple infrequent WT-MetaD simulations to estimate more precisely all exchange steps. Applying the same setup of Section 4.3, we simulated two mechanisms of exchange (Supplementary Figure 12a). In the first one, we activated the **Mn** monomer exchange from the tip of the **Zn** fiber directly out in solution (A->C: 1-step transition). In this case, we monitored in each infrequent WT-MetaD run, the time at which the number of contacts between the **Mn**-monomer and all other monomers in the **Zn**-fiber drops to 0 (from stacked to exchanged out the fiber). For the second mechanism, we estimated the two transitions A->B and B->C (2-steps transition). For the A->B transition, we monitored the time, in each infrequent WT-MetaD run, at which the number of contacts between the **Mn**-monomer core and the other cores in **Zn**-fiber drops to 0 (from stacked to adsorbed configuration). For the B->C transition, we started from a configuration where the **Mn** monomer is adsorbed onto the **Zn**-fiber surface and activated its detachment and jumping in solution in multiple infrequent WT-MetaD simulations (monitoring the time at which the number of contacts between the adsorbed **Mn**-monomer and all other monomers in the **Zn**-fiber drops to 0). Supplementary Figure 12c shows all the collected transition time distributions, and the related Poissonian fits. From these, we estimated the characteristic transition timescales for all transitions (Supplementary Figure 12c: colored dots on the *x* axis).

The Poissonian distributions allowing to estimate the characteristic kinetics of the rare exchange events A to B (violet), B to C (orange) and A to C (blue) transitions. For the A->C transition (blue line) we found a transition time of the CG model of ~10^0 s. This timescale is in the same order of magnitude with respect to the one found for the disruption of the **Mn-Mn** dimer (Fig. 4c of the main paper), which is consistent with the very similar **Mn**-**Mn** and **Mn**-**Zn** core-core dimerization free-energies (see main paper). When comparing the 1-step (A->C) to the 2-steps (A->B + B->C) exchange mechanisms, we observe that in this case the characteristic timescale for the A->B transition is again very similar to that of A->C (see Supplementary Figure 12c: blue and violet Poissonian fits). The free energy barriers for the A->B and A->C transitions are very similar. This means that in the **Mn**-monomer exchange, the A->B transition is the rate limiting step: the strong directional interaction between the porphyrin cores (**Mn** onto **Zn**) makes the transition from the stacked-tip (A) to an adsorbed (B) configuration a rare event. The reverse B to A transition requires crossing a much lower barrier (Supplementary Figure 12b). The characteristic timescale for the B->C transition is much lower (Supplementary Figure 12c, orange: ~10^{-6} s). Once a **Mn**-monomer is adsorbed onto the fiber surface (state B), jumping in solution is a much easier step (C), as it is reasonable for substantially solvophilic monomers.

Altogether, these results indicate that (i) in exchanging out from a **Zn**-fiber tip, a **Mn**-monomer first breaks the directional stacking interaction with the closest monomer core in the fiber (rate limiting

step), and after this, it rapidly jumps in solution. Conversely, (ii) in being incorporated withing the **Zn**-fiber from the solution, the **Mn**-monomer first impacts on the **Zn**-fiber surface (most probable event considered the aspect ratio of these fibers), and then it reaches the fiber tip being incorporated into the stack. Complete details of all molecular models used for the simulations, and of the simulation parameters (input files, etc.) are available at https://doi.org/10.5281/zenodo.5717968.

2.4 Dilution-induced supramolecular polymerization

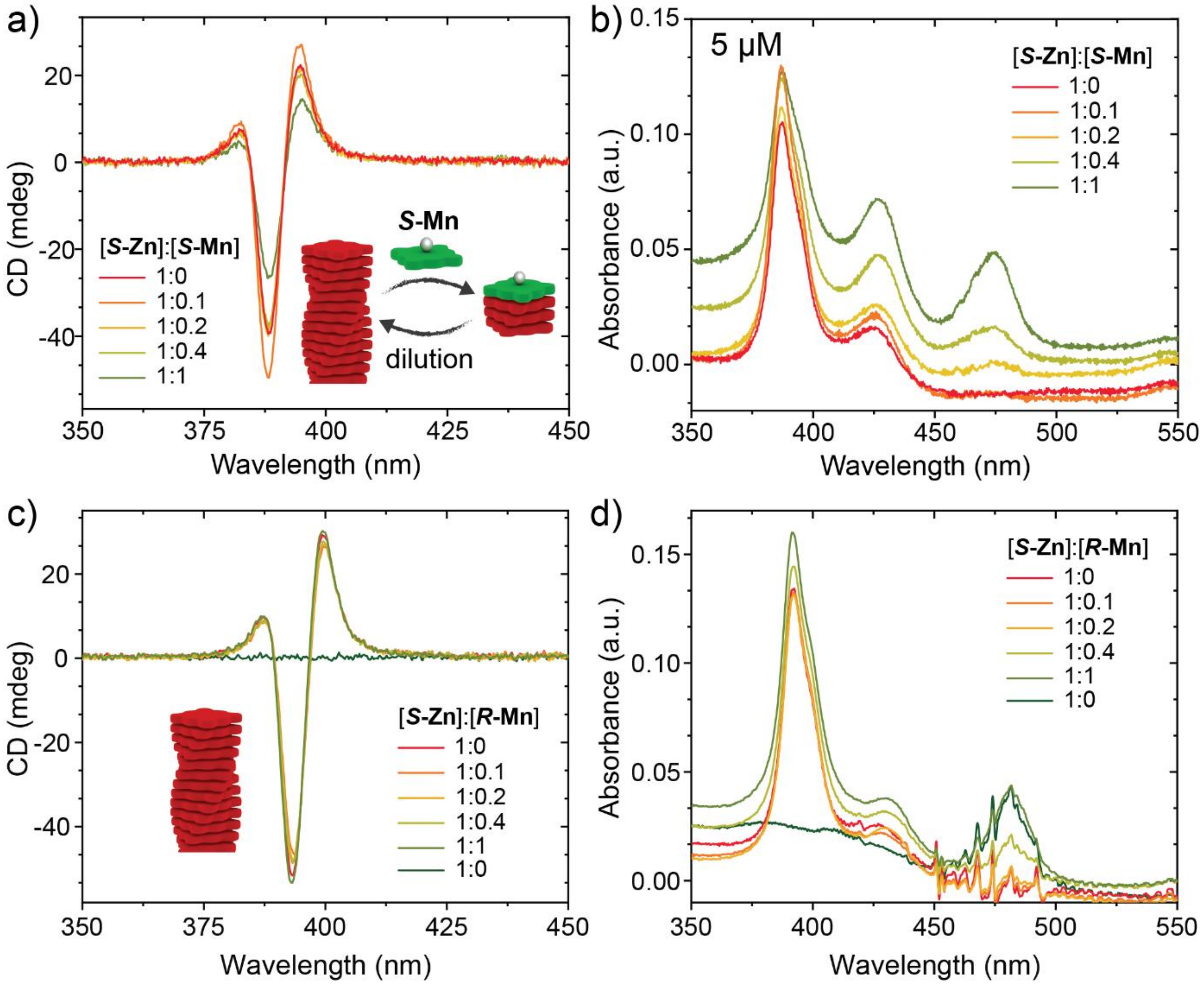

Supplementary Figure 13: a, c) CD and b, d) absorbance spectra showing the presence of chiral H-aggregates in all mixtures of ***S*-Zn** with increasing molar ratios of ***S/R*-Mn** at dilute concentrations of $c_{S\text{-}Zn}$ = 5 µM in MCH.

Atomic force microscopy (AFM)

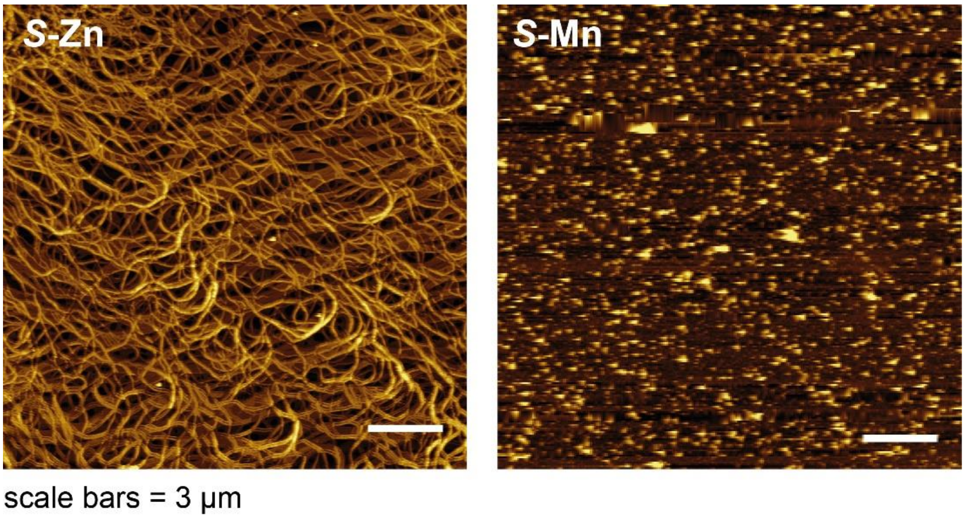

Supplementary Figure 14: AFM images of ***S*-Zn** and ***S*-Mn** dropcasted from 25 µM solutions in MCH on Mica. Scale bars represent 3 µm.

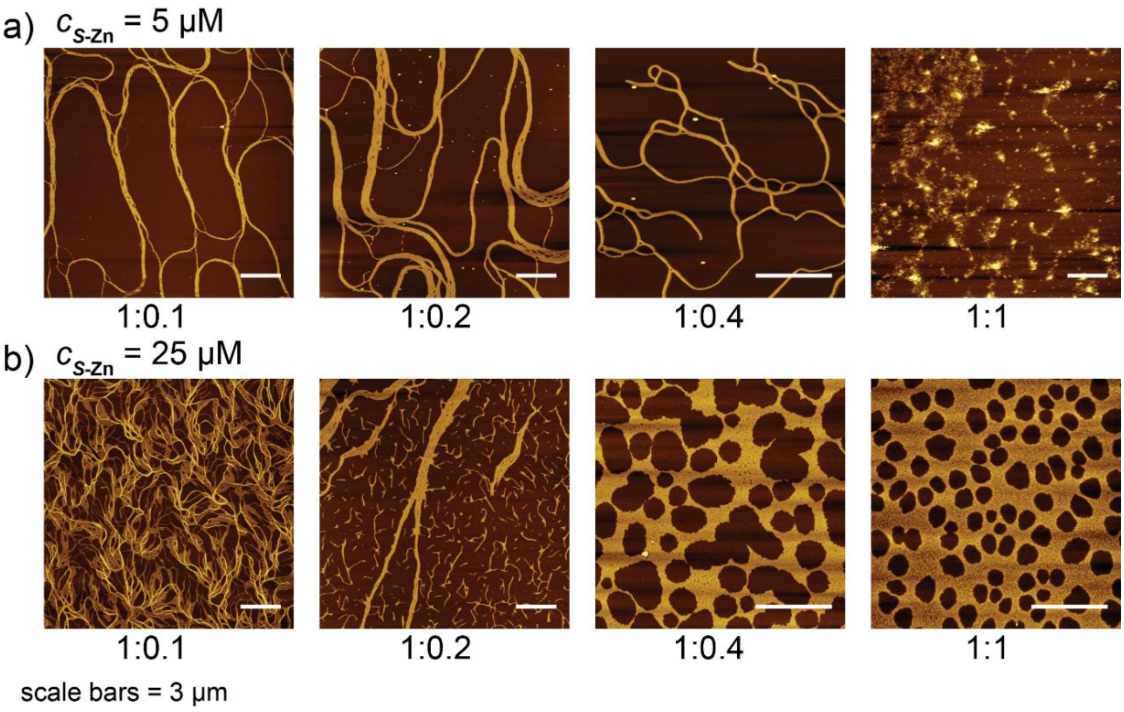

Supplementary Figure 15: AFM microscopy images of mixtures of ***R*-Mn** and ***S*-Zn** at a) 5 µM and b) 25 µM concentration of ***S*-Zn**. Scale bars represent 3 µm in all images.

Kinetics of aggregate equilibration

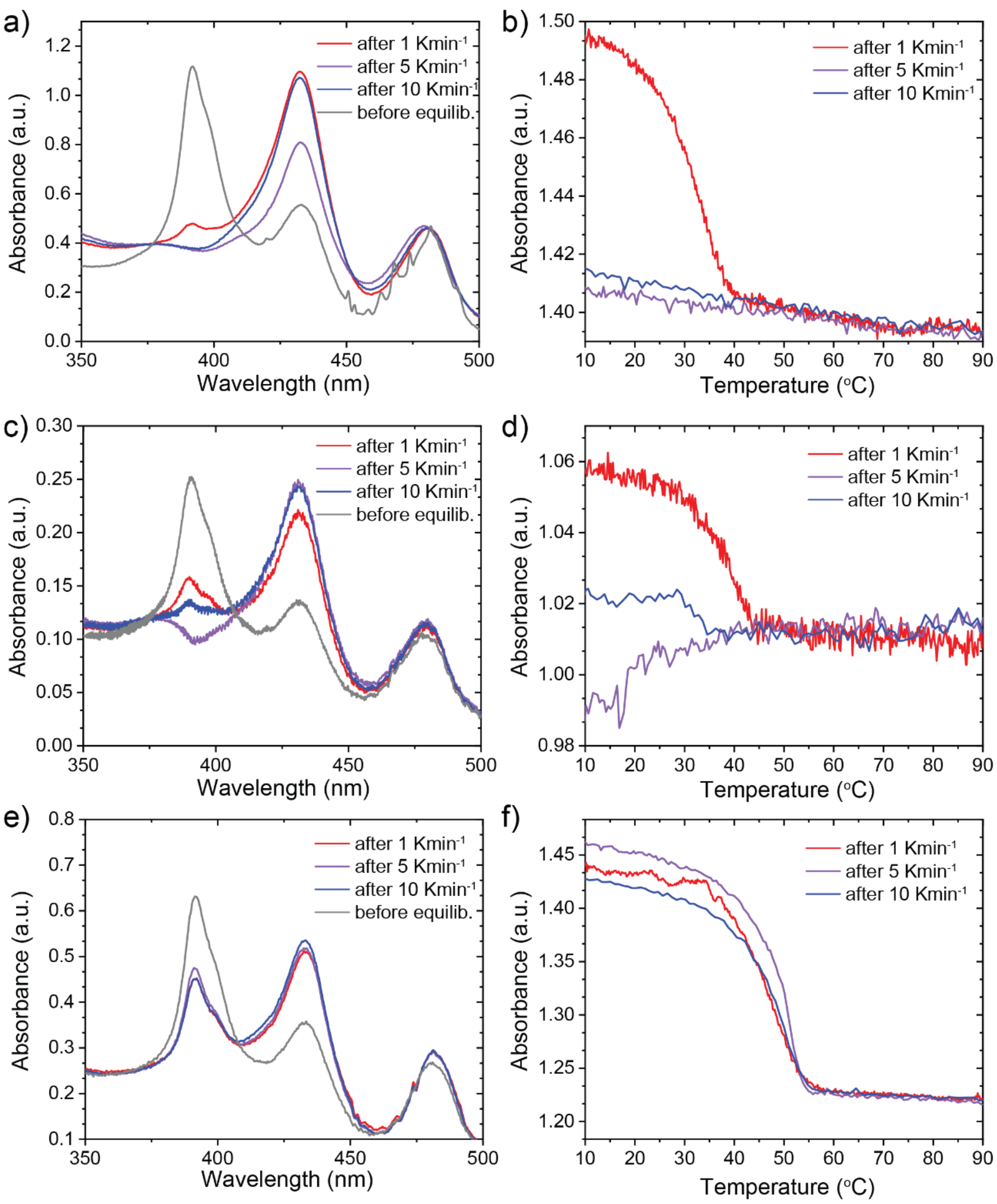

Supplementary Figure 16: Kinetic influence on equilibrium between aggregates. Full absorbance spectra and melting curves for a 1:1 mixture of [*S*-Mn]:[*S*-Zn] at c = 5 µM (a, b), 10 µM (c, d) and 25 µM (e, f) in MCH, cooling rates are indicated in the plots, relative changes in absorbance for b, d and f were monitored at λ = 390 nm.

2.5 Spectroscopic data of compounds

MALDI-TOF of ***S***-Mn and ***R***-Mn:

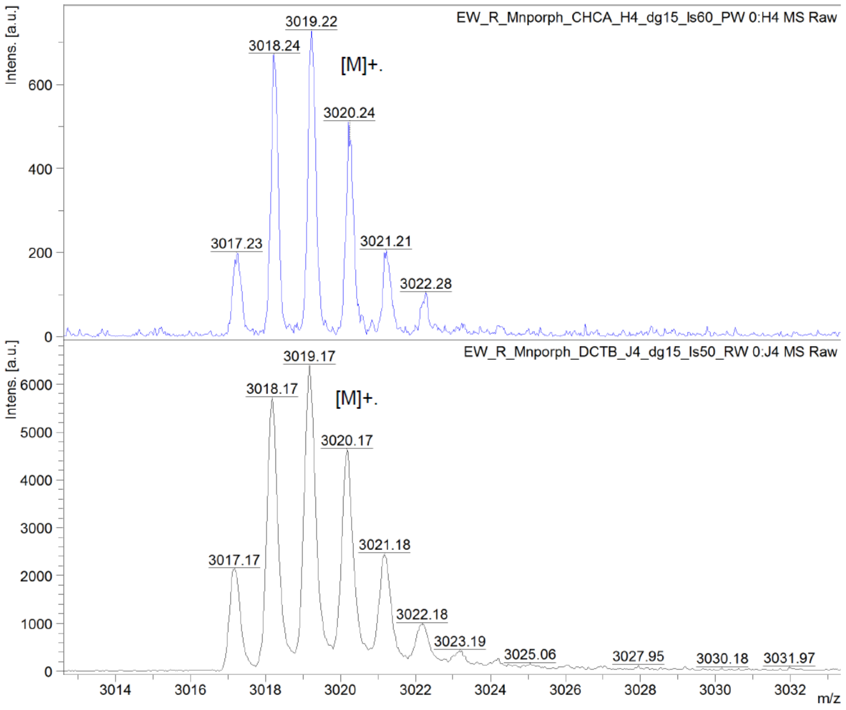

Supplementary Figure 17: MALDI spectrogram of *R*-Mn.

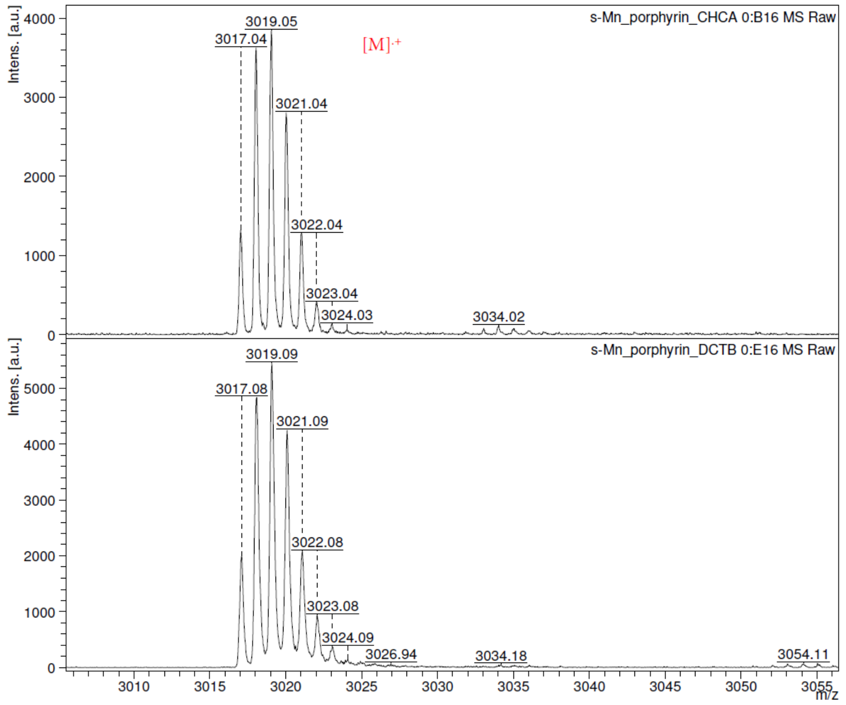

Supplementary Figure 18: MALDI spectrogram of *S*-Mn.

3. Supplementary References

1. Helmich, F., Smulders, M. M. J., Lee, C. C., Schenning, A. P. H. J. & Meijer, E. W. Effect of stereogenic centers on the self-sorting, depolymerization, and atropisomerization kinetics of porphyrin-based aggregates. *J. Am. Chem. Soc.* **133**, 12238–12246 (2011).

2. Helmich, F. *et al.* Dilution-induced self-assembly of porphyrin aggregates: A consequence of coupled equilibria. *Angew. Chemie Int. Ed.* **49**, 3939–3942 (2010).

3. Helmich, F. A. Multi-component porphyrin self-assembly. (Eindhoven University of Technology, 2012). doi:10.6100/IR723515.

4. Jung, S. H., Bochicchio, D., Pavan, G. M., Takeuchi, M. & Sugiyasu, K. A Block Supramolecular Polymer and Its Kinetically Enhanced Stability. *J. Am. Chem. Soc.* **140**, 10570–10577 (2018).

5. Wang, J., Wolf, R. M., Caldwell, J. W., Kollman, P. A. & Case, D. A. Development and testing of a general Amber force field. *J. Comput. Chem.* **25**, 1157–1174 (2004).

6. Bayly, C. I., Cieplak, P., Cornell, W. D. & Kollman, P. A. A well-behaved electrostatic potential based method using charge restraints for deriving atomic charges: The RESP model. *J. Phys. Chem.* **97**, 10269–10280 (1993).

7. Marrink, S. J., Risselada, H. J., Yefimov, S., Tieleman, D. P. & De Vries, A. H. The MARTINI force field: Coarse grained model for biomolecular simulations. *J. Phys. Chem. B* **111**, 7812–7824 (2007).

8. Sarkar, A. *et al.* Self-Sorted, Random, and Block Supramolecular Copolymers via Sequence Controlled, Multicomponent Self-Assembly. *J. Am. Chem. Soc.* **142**, 7606–7617 (2020).

9. Casellas, N. M. *et al.* From isodesmic to highly cooperative: reverting the supramolecular polymerization mechanism in water by fine monomer design. *Chem. Commun.* **54**, 4112–4115 (2018).

10. Bochicchio, D. & Pavan, G. M. From Cooperative Self-Assembly to Water-Soluble Supramolecular Polymers Using Coarse-Grained Simulations. *ACS Nano* **11**, 1000–1011 (2017).

11. Barducci, A., Bussi, G. & Parrinello, M. Well-tempered metadynamics: A smoothly converging and tunable free-energy method. *Phys. Rev. Lett.* **100**, 020603 (2008).

12. Berendsen, H. J. C., van der Spoel, D. & van Drunen, R. GROMACS: A message-passing parallel molecular dynamics implementation. *Comput. Phys. Commun.* **91**, 43–56 (1995).

13. Bonomi, M. *et al.* Promoting transparency and reproducibility in enhanced molecular

simulations. *Nature Methods* vol. 16 670–673 (2019).

14. Tribello, G. A., Bonomi, M., Branduardi, D., Camilloni, C. & Bussi, G. PLUMED 2: New feathers for an old bird. *Comput. Phys. Commun.* **185**, 604–613 (2014).

15. Parrinello, M. & Rahman, A. Polymorphic transitions in single crystals: A new molecular dynamics method. *J. Appl. Phys.* **52**, 7182–7190 (1981).

16. Essmann, U. *et al.* A smooth particle mesh Ewald method. *J. Chem. Phys.* **103**, 8577–8593 (1995).

17. Bussi, G., Donadio, D. & Parrinello, M. Canonical sampling through velocity rescaling. *J. Chem. Phys.* **126**, (2007).

18. Berendsen, H. J. C., Postma, J. P. M., Van Gunsteren, W. F., Dinola, A. & Haak, J. R. Molecular dynamics with coupling to an external bath. *J. Chem. Phys.* **81**, 3684–3690 (1984).

19. Tiwary, P. & Parrinello, M. From metadynamics to dynamics. *Phys. Rev. Lett.* **111**, 230602 (2013).

20. Salvalaglio, M., Tiwary, P. & Parrinello, M. Assessing the reliability of the dynamics reconstructed from metadynamics. *J. Chem. Theory Comput.* **10**, 1420–1425 (2014).
